# The anterior gradient homologue 2 (AGR2) co-localises with the glucose-regulated protein 78 (GRP78) in cancer stem cells, and is critical for the survival and drug resistance of recurrent glioblastoma: in situ and in vitro analyses

**DOI:** 10.1186/s12935-022-02814-5

**Published:** 2022-12-08

**Authors:** Deema Hussein, Reem Alsereihi, Abdulla Ahmed A. Salwati, Rinad Algehani, Alazouf Alhowity, Ahmed M. Al-Hejin, Hans-Juergen Schulten, Saleh Baeesa, Mohammed Bangash, Fahad Alghamdi, Richard Cross, Torki Al Zughaibi, Mohamad Saka, Adeel Chaudhary, Adel Abuzenadah

**Affiliations:** 1 King Fahd Medical Research Center, King Abdulaziz University, 80216, Jeddah, 21589 Saudi Arabia; 2 Department of Medical Laboratory Sciences, Faculty of Applied Medical Sciences, King Abdulaziz University, Jeddah, 21589 Saudi Arabia; 3 Department of Biological Sciences, Faculty of Science, King Abdulaziz University, 80203, Jeddah, 21589 Saudi Arabia; 4 College of Health Sciences, Al-Rayan Colleges, 41411, Madinah AL-Munawarah, Saudi Arabia; 5 Center of Excellence in Genomic Medicine Research, Department of Medical Laboratory Technology, Faculty of Applied Medical Sciences, King Abdulaziz University, Jeddah, 21589 Saudi Arabia; 6 Division of Neurosurgery, Faculty of Medicine, King Abdulaziz University, Jeddah, 21589 Saudi Arabia; 7 Pathology Department, Faculty of Medicine, King Abdulaziz University, Jeddah, 21589 Saudi Arabia; 8 School of Engineering and Sustainable Development, Emerging Technologies Research Centre (EMTERC), De Montfort University, The Gateway, Leicester, LE1 9BH UK; 9 Centre of Innovation for Personalized Medicine, Department of Medical Laboratory Technology, Faculty of Applied Medical Sciences, King Abdulaziz University, Jeddah, 21589 Saudi Arabia

**Keywords:** Glioblastoma, Cancer stem cells (CSCs), Anterior gradient homologue 2 (AGR2), Glucose-regulated protein 78 (GRP78), Drug resistance

## Abstract

**Background:**

Glioblastomas (GBs) are characterised as one of the most aggressive primary central nervous system tumours (CNSTs). Single-cell sequencing analysis identified the presence of a highly heterogeneous population of cancer stem cells (CSCs). The proteins anterior gradient homologue 2 (AGR2) and glucose-regulated protein 78 (GRP78) are known to play critical roles in regulating unfolded protein response (UPR) machinery. The UPR machinery influences cell survival, migration, invasion and drug resistance. Hence, we investigated the role of AGR2 in drug-resistant recurrent glioblastoma cells.

**Methods:**

Immunofluorescence, biological assessments and whole exome sequencing analyses were completed under in situ and in vitro conditions. Cells were treated with CNSTs clinical/preclinical drugs taxol, cisplatin, irinotecan, MCK8866, etoposide, and temozolomide, then resistant cells were analysed for the expression of AGR2. AGR2 was repressed using single and double siRNA transfections and combined with either temozolomide or irinotecan.

**Results:**

Genomic and biological **c**haracterisations of the AGR2-expressed Jed66_GB and Jed41_GB recurrent glioblastoma tissues and cell lines showed features consistent with glioblastoma. Immunofluorescence data indicated that AGR2 co-localised with the UPR marker GRP78 in both the tissue and their corresponding primary cell lines. AGR2 and GRP78 were highly expressed in glioblastoma CSCs. Following treatment with the aforementioned drugs, all drug-surviving cells showed high expression of AGR2. Prolonged siRNA repression of a particular region in AGR2 exon 2 reduced AGR2 protein expression and led to lower cell densities in both cell lines. Co-treatments using AGR2 exon 2B siRNA in conjunction with temozolomide or irinotecan had partially synergistic effects. The slight reduction of AGR2 expression increased nuclear Caspase-3 activation in both cell lines and caused multinucleation in the Jed66_GB cell line.

**Conclusions:**

AGR2 is highly expressed in UPR-active CSCs and drug-resistant GB cells, and its repression leads to apoptosis, via multiple pathways.

**Supplementary Information:**

The online version contains supplementary material available at 10.1186/s12935-022-02814-5.

## Introduction

Glioblastomas are characterised as grade 4, and are highly aggressive primary central nervous system tumours (CNSTs) [1]. Tumours are often devastating, and they have a poor prognosis and a limited survival time of approximately 2.4 years [2, 3]. The optimal treatment for glioblastomas is complete resection through surgery. However, for inoperable tumours, other treatments are used, including radiation and cytotoxic chemotherapy. For incomplete surgical resection, a median survival of 12–15 months after diagnosis, and a less than 5% of patients surviving more than 5 years have been reported [4].

A whole bulk DNA analysis of glioblastoma tissues led to the identification of mutations in isocitrate dehydrogenase 1/2 *(IDH*1/2), phosphatase and tensin homolog (*PTEN*), cyclin dependent kinase inhibitor 2A (*CDKN2A*), tumour protein p53 (*TP53*), parkin RBR E3 ubiquitin protein ligase (*Park2*), protein tyrosine phosphatase receptor type D (*PTPred*) and neurofibromin 1(*NF1*) genes. Several amplifications of genes have also been identified including the amplifications of the epidermal growth factor receptor (*EGFR*), fibroblast growth factor receptor 2 (*FGFR2*), insulin receptor substrate 2 (*IRS2*), and AKT serine/threonine kinase 3 (*AKT3*) [5]. In contrast, single-cell sequencing experiments highlighted a high level of inter-and intra-patient heterogeneity [6, 7]. Further work supported vast differences in the genetic profiles of different glioblastoma patients and showed a remarkably high plasticity between different cell types within a tumour [8, 9].

Single cell sequencing analysis also identified a high heterogeneity of cancer stem cells (CSCs) within glioblastomas [7, 9]. CSCs are cancer cells that utilise stem cell pathways to promote tumour growth and are linked with aggressiveness and treatment resistance [10, 11]. Stem cell associated pathways have recently been classified as a new hallmark of cancer referred to as “Unlocking Phenotypic Plasticity” [12]. Glioblastoma CSCs express numerous markers [13, 14]. However, a single marker is insufficient to indicate the presence of CSCs, rather it is the expression of multiple CSC markers that is indicative of the presence of these cells [14, 15].

The unfolded protein response (UPR) machinery resides in the endoplasmic reticulum (ER) and is responsible for the degradation of misfolded proteins. UPR is triggered in normal cells via the increased expression of ER proteins and their localisation to the ER [16]. In cancer cells, this machinery is deregulated as the accumulation of misfolded/unfolded proteins increases greatly [17]. Glucose-regulated protein 78 (GRP78), also known as heat shock protein A5 (HSPA5) or binding immunoglobulin protein (BIP), is a critical UPR marker that acts as an ER molecular chaperone and modulates the UPR machinery [18]. GRP78 was found to be highly expressed in glioblastomas [19, 20], and several studies have demonstrated that its increased expression enhances cell survival, migration, invasion, and drug resistance [21–23].

A partner of the GRP78 protein is the anterior gradient homologue 2 (AGR2), which was shown to inhibit the UPR-endoplasmic reticulum associated protein degradation (ERAD) process [24]. The *AGR2* gene is located in chromosome7p21.3; it has 8 exons, and transcribes six splice variants: AGR2vC, AGR2vD, AGR2vE, AGR2vF, AGR2vG, and AGR2vH [25]. AGR2 is highly expressed in the stomach, colon and duodenum; however, it is not expressed in normal brain tissues [26, 27]. Knockout experiments in mice showed that AGR2-/- mice poorly develop inner colon mucus layers [28], have deformed tissue regeneration [29], and develop severe acute terminal ileitis with multinucleated giant cells reminiscent of granulomatous inflammation [30]. In cancer cells, the protein was shown to be upregulated in several aggressive types including glioblastoma [31, 32], and it was found to colocalise with CSC markers in high-grade tumours [31, 33–35]. Secreted AGR2vH, which can be detected in the blood and urine of cancer patients, was shown to contribute to metastasis [15, 36–38].

To date, the contribution of GRP78 and AGR2 to drug resistance in CSCs of glioblastoma tissues and primary cell lines has not been clarified. In the present study, we determined the levels of the AGR2 protein expression in recurrent glioblastoma tissues and primary cell lines, and explored its co-expression with GRP78 and several CSC markers. We further investigated the relationship between AGR2 expression and drug resistance for clinically/preclinically known chemotherapeutics, and assessed the effects on cell survival upon repression of AGR2 using small interfering RNA (siRNA) sequences in combination with chemotherapeutic drugs.

## Methods

### Tissue and cell line retrieval

The examined tissues and cell lines were recurrent primary glioblastoma tumours obtained from the Neurooncology Translational Group at King Fahad Medical Research Center for patients who were treated at King Abdulaziz University Hospital. The Jed66_GB cell line was retrieved from a recurrent adult-type glioblastoma, IDH1/2-wild type, World Health Organisation (WHO) grade 4, from a 32-year-old male. The Jed41_GB cell line was retrieved from a recurrent tumour of a 5-year-old boy diagnosed with glioblastoma, giant cell variant, IDH1/2-wild type, WHO grade 4, Additional file 1: Figure S1 (Data retrieved from whole exome sequencing showed no damaging variants for IDH1/2). The samples were recovered and cultured as previously described [39]. For all experiments, early passages up to passage 35 were used to ensure that both cell line models remained genomically similar to the original tumours.

### Biological assessments

To assess the clonogenic capacity, cells were plated in 6-well plates at different cell numbers and were cultured to form clones. 14 to 21 days following seeding, clones were fixed with 4% paraformaldehyde (PFA), then stained with crystal violet, and counted. All experiments were carried out in biological triplicate. To track migratory progression, scratch assay experiments were performed as previously described [39]. Briefly, cells were plated in 6-well plates, left to adhere overnight and a scratch ‘wound’ was made using a 10 µl pipette tip when the cells reached 80% confluency, and cells’ movements were followed up for 3–4 days. Cell monitored at 5 × magnification using a digital microscope camera (Leica DFC425).

### DNA whole exome and conventional sequencing of tissue and cell lines

DNA was extracted from the tissues and corresponding cell lines (Jed66_GB cell line passage 22 and Jed41_GB cell line passage 25) (DNeasy Blood & Tissue Kit, 69504, Qiagen), and whole exomes were sequenced using Agiomix, UAE services. VCF files were annotated using the BaseSpace Variant Interpreter (Illumina, San Diego, CA) (accessed on the 05/12/2021). Only those COSMIC variants that had damaging coding consequences were further assessed, as called by BaseSpace Variant Interpreter, or checked manually using PolyPhen-2 Wiki. They had a population frequency of less than 0.01 for all population sources. COSMIC variants detected in the tissues and corresponding cell lines were selected to enrich the tumorigenic and progressive genes. One critical exon variant in *STC2* in Jed66_GB and one in *TP53* in Jed41_GB were assessed by conventional sequencing that was performed on tissue and corresponding cell lines as described in our standard protocol [40]. The primers used for PCR amplification and sequencing were for *STC2*-exon 4 [forward and reverse primer sequences (3′-5′): TGAGCGAGGTAGCAAGAG and CGATGAAGTCCACAGTCC]; and *TP53* in intron 8-exon9 [forward and reverse primer sequences (3′-5′): TTCCTTACTGCCTCTTGC, and GCTTCTTGTCCTGCTTGC]. The specificity of the PCR products was ascertained by gel electrophoresis. Subsequently, the PCR products were purified and underwent cycle sequence reactions using a BigDye Terminator V3.1 Cycle Sequencing Kit (Thermo Fisher Scientific Inc., Waltham, MA). The purified sequencing products were finally resolved with capillary electrophoresis (3130 Genetic Analyzer, Thermo Fisher Scientific Inc., Massachusetts, USA).

### Immunofluorescence staining

To process in situ immunofluorescence, tissue sections were fixed and stained as previously described [15]. For the cell line immunofluorescence staining, the cells were seeded on chamber slides, fixed and stained as previously described [39]. The rabbit primary antibodies used in this study were anti-AGR2 (1:100, ab76473, raised against AGR2 exon 2, abcam), anti-Ki67 (1:200, ab16667, abcam), anti-SRY-box transcription factor 2 (SOX2) (1:200, 09–0024, Miltenyi), anti-Frizzled 9 (FZD9) (1:100, ab150515, abcam), anti-glial fibrillary acidic protein (GFAP) (1:500, ab7260, abcam), anti-oligodendrocyte transcription factor 2 (OLIG2) (1:500, ab42453, abcam), anti-tubulin beta 3 class III (TUBB3) (1:100, ab18207, abcam), and anti-Caspase-3 (1:100, ab40a51, abcam). The mouse primary antibodies used in this study were anti-GRP78 (1:50, ab181499, abcam), anti-BMI1 proto-oncogene, polycomb ring finger (BMI1) (1:100, ab14389, abcam), anti-Nestin (1:50, ab6142, abcam), anti-Vimentin (VIM) (1:100, ab8978, abcam), anti-stage-specific embryonic antigen-4 (SSEA4) (1:100, ab16287, abcam), anti-prominin 1 (CD133) (1:100, W6B3C1, Miltenyi), and anti-tumour protein p53 (P53) (1:100, ab26, abcam). For secondary goat antibodies, 488 anti-mouse (1:300, ab150105, abcam) and 555 anti-Rabbit (1:700, ab150074, abcam) antibodies were used. Vectashield with DAPI was added to stain the nuclei. Pictures were taken at 20 × magnification using a Leica DMI6000 microscope and a Leica DFC425 camera and they were processed as previously described [15, 39].

### Clonogenic cytotoxic assay and growth inhibition

Several drugs were used in this study. Taxol, a microtubule stabiliser (T7402, Sigma, dissolved in DMSO); cisplatin, a DNA cross-linker (P4394, Sigma, dissolved in saline); irinotecan, a DNA topoisomerase I inhibitor (I1406, Sigma, dissolved in DMSO); MCK8866, IRE1α inhibitor (HY-104040, MCE, dissolved in water); etoposide, DNA topoisomerase II inhibitor (E1383, Sigma, dissolved in DMSO); and temozolomide, DNA guanine and adenine methylation agent (HY-17364, MCE, dissolved in DMSO).

For the clonogenic cytotoxic assay, cells were plated in 6-well plates at 1,000 and 2,000 cells per well for Jed66_GB and Jed41_GB, respectively. Cells were supplemented with DMEM-F12 and 10% FBS and left to adhere overnight at 37 °C with 5% CO_2_. On the next day, cells were treated for two hours with either cisplatin or etoposide, or for three days with either taxol, or irinotecan or MCK8866. 14 to 21 days following seeding, the clones were fixed with 4% PFA, stained with crystal violet and counted.

To determine the IC_50_ values for temozolomide and irinotecan in order to use for growth inhibition assays, cells were plated in 96-well plates at 5 × 10^3^ or 1 × 10^4^ cells/well for Jed66_GB and Jed41_GB, respectively. For temozolomide, the concentrations used were 50, 100, 200, 400, 800, 1600, and 3200 µM for both cell lines. For irinotecan the concentrations 0.5, 1, 2, 4, 8, and 16 µM were used to treat Jed66_GB, while for Jed41_GB higher concentrations were required (25, 50, 100, 200, 400, 800 μM). After three days of drugs incubation, the cells were fixed with 4% PFA, stained with 1% crystal violet, and the absorbance was measured at 595 nm. All assays were performed in triplicate.

### siRNA transfections

For basic transfection optimisation, cells were seeded in 8-well chamber slides at 2 × 10^4^ cells/well and 4 × 10^4^ cells/well for Jed66_GB, and Jed41_GB, respectively and were left overnight to attach in 400μL Transfection Media (TM) (DMEM-F12 media with 10% FBS and no antibiotics). The following day, cells were transfected with 0.6% or 1.0% of lipofectamine (Lipofectamine®RNAiMAX, 13778075, Integrated DNA Technology), or with 0.2% or 0.3% of Cy3-DsiRNA (CY3 DS Transfection Control, 77619141, Integrated DNA Technology), or with 0.2% or 0.3% of the negative control (DsiRNA-Negative Control, 77619142, Integrated DNA Technology). Cell death was at 80% using 1% lipofectamine concentration in initial experiments; therefore, the concentration of 0.6% lipofectamine was used for further AGR2 siRNA experiments.

To optimise AGR2 repression using a single transfection, three siRNAs targeting different exon sequences were tested at a final concentration of 0.2% (Integrated DNA Technologies, Coralville, IO). The siRNA sequences were as follows: AGR2 exon 2A siRNA (5′-AGAUACCACAGUCAAACCUGGAGCCAAA-3′) (hs.Ri.AGR2.13.5, Integrated DNA Technology), AGR2 exon 2B siRNA (5′-AAAGGACUCUCGACCCAAACUGCCCC-3′) (Customised, Integrated DNA Technology) and AGR2 exon 5/6 siRNA (5′- CUGGUUUAUGAAACAACUGACAAACAC- 3′) (hs.Ri.AGR2.13.8, Integrated DNA Technology). The cells were seeded as previously mentioned; then they were transfected, fixed with 4% PFA after 24/48 h of incubation and processed for immunofluorescence staining. For double transfections, cells were seeded as previously mentioned, transfected with control siRNA or AGR2 exon 2B siRNA, fixed with 4% PFA after two weeks and processed for immunofluorescence staining.

To determine the siRNA-treatment-period that is appropriate to detect a possible early repression of AGR2, before any signs of cell death or reduction in cell densities were observed, cells were seeded and transfected with control siRNA or AGR2 exon 2B siRNA, as previously mentioned. The most appropriate post-transfection incubation periods were then determined to be eight hours for Jed66_GB cells, and 36 h for Jed41_GB. The fixation process and immunofluorescence staining were then carried out in the manner described previously.

### siRNA transfections combined with drug treatments

For combined transfections and drug treatments, cells were incubated in 96-well plates in 200μL of TM/well at 5 × 10^3^ or 1 × 10^4^ cells/well for Jed66_GB and Jed41_GB, respectively. Cells were either exposed to TM, or transfected with AGR2 exon 2B siRNA as mentioned above. After 24/48 h, the TM was removed, and either DMASO (1%), temozolomide (IC_50_ at 700 µM for Jed66_GB and 1000 µM for Jed41_GB) or irinotecan (IC_50_ at 2.05 µM for Jed66_GB and 100 µM for Jed41_GB) were added. After three days, the cells were fixed and stained using 1% crystal violet with 10% acetic acid, then the absorbance was measured at 595 nm. The combined transfection and drug treatments were also carried out in chamber slides in parallel to assess AGR2 expression via immunofluorescence staining.

### Assessment of AGR2 repression and cell densities

To assess cell density, the number of nuclei per 3.9 mm^2^ was counted using ImageJ. The average number of untreated cells per 3.9 mm^2^ was considered as a cell density of 100% and served to calculate the cell density percentage for the average number of treated cells per 3.9 mm^2^.

To assess AGR2 repression in individual cells that were immunofluorescence-co-stained for anti-AGR2 and anti-GRP78, images were analysed using ImageJ. Individual cells were selected using the polygon selection tool, and data for mean area intensities were recorded using the analyse, histogram and RGB tools. At least 100 cells were counted. P53 nuclear expression was assessed in at least 100 cells. All assays were performed in triplicates.

### Statistical analysis of the data

The results were analysed using SPSS version 21.0. T-test, Z-test or Chiχ^2^ test were used to assess for significant differences in the mean percentage of cell density, survival fractions, areas' intensities and the presence of multinucleated cells.

## Results

### Basic characteristics of the AGR2-expressed glioblastoma primary cell lines

The two primary Jed66_GB and Jed41_GB cell lines showed a high expression of AGR2, which was detected in both the nucleus and the cytoplasm (Additional file 2: Figure S2A). The primary cell lines had high proliferation rates in culture (doubling times of 5 ± 1.3 days and 8 ± 1.9 days, respectively) and had the ability to form clones (1000 cells initiated an average of 126 clones for Jed66_GB and 43 clones for Jed41_GB after two to three weeks of incubation) (Additional file 2: Figure S2B, C). Both cell lines had medium positive staining for the proliferation marker Ki67 (an average of 47.9 ± 7.9% and 15.5 ± 4.7%, respectively) and a high positive staining for the cancer biomarker BMI1 (an average of 82.7% and 64.6%, respectively) (Additional file 2: Figure S2D, E). Both cell lines were capable of cell migration as indicated by the scratch assay (Additional file 2: Figure S2F). The cell lines and tissues were whole exome sequenced and rare damaging COSMIC variants present in each cell line and its corresponding tissue (TC) were identified (Additional file 6: Tables S1 and Additional file 7: Table S2). No *IDH*-damaging variants were detected. Two critically damaging variants, the missense variant p.Glu293Lys in the stanniocalcin-2 *(STC2*) gene detected in Jed66_GB and the homozygous missense variant p.Arg273Cys in *TP53* detected in Jed41_GB, were conventionally sequenced, and the results concurred with those detected via whole exome sequencing (Additional file 3: Figure S3A). Neither *AGR2* nor *GRP78* contained critical variants in the cell lines nor in the tissues, and only non-damaging and common variants in these genes were detected (Additional file 3: Figure S3B). The critical functions likely to be affected in the studied cell lines, as per related TC-rare damaging COSMIC variants, are shown in Additional file 8: Table S3. Prominent functions common to both cell lines are shown in Additional file 3: Figure S3C.

### AGR2 colocalises with the UPR marker GRP78 in glioblastoma tissues and corresponding primary cell lines

**Fig. 1:**
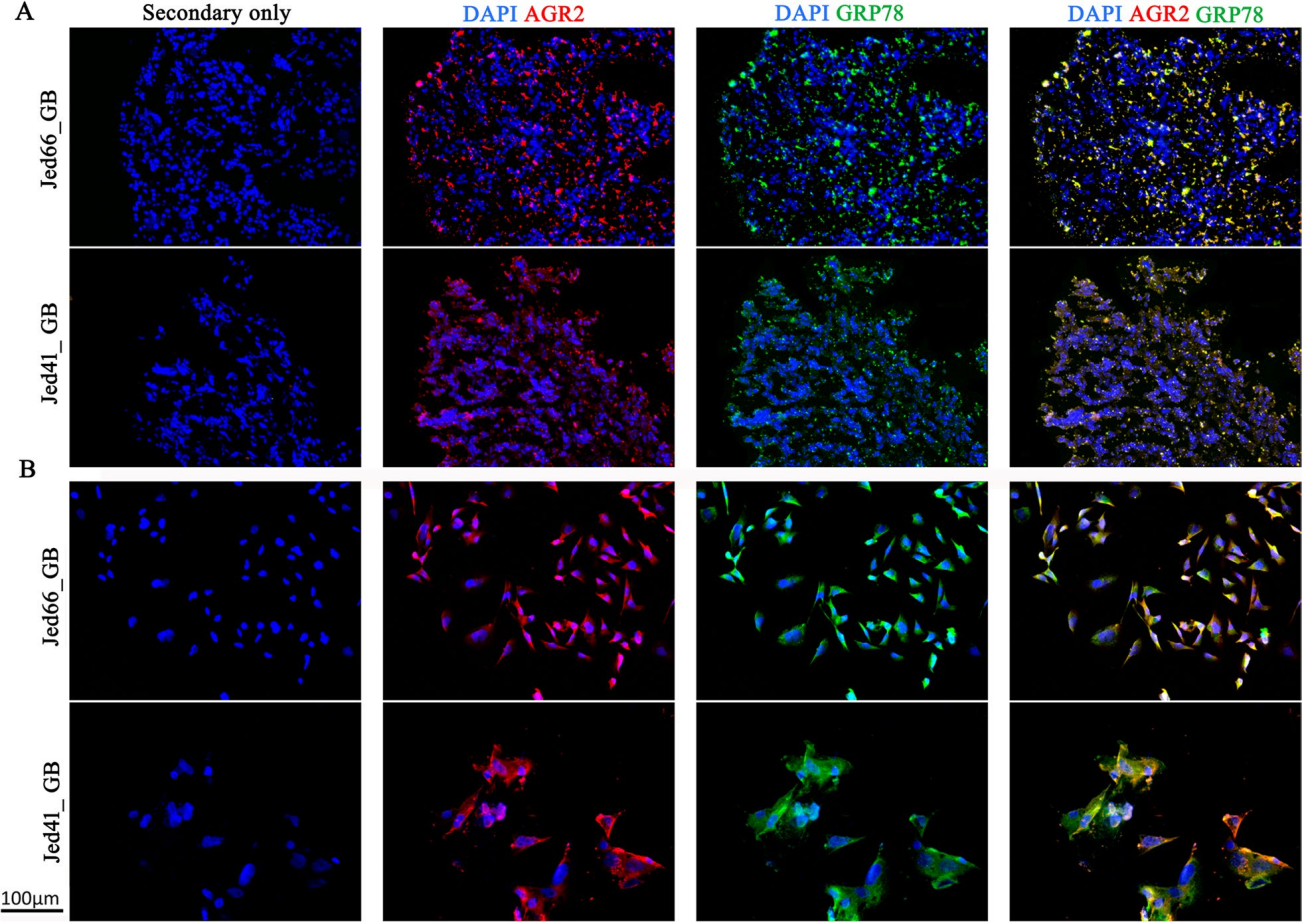
AGR2 localises with the UPR marker GRP78 in the glioblastoma tissues and corresponding cell lines Jed66_GB and Jed41_GB. Immunofluorescence images for AGR2 (red) co-localising with the GRP78 (green) in **A**) fresh frozen tissues and **B**) primary corresponding cell lines. DAPI is shown in blue. All images were taken at 20 × magnification

To ensure that the detected expression of AGR2 in both cell lines was not a superficial phenomenon related to cell culturing, the corresponding tissues that were used to generate the primary cell lines were also stained to detect AGR2. Almost all cells in the tissues expressed AGR2 (Fig. 1). To investigate whether AGR2 was co-expressed with its associated partner, the UPR marker GRP78 in the studied samples, AGR2 and GRP78 staining was examined in both tissues and the corresponding cell lines (Fig. 1A and B). The images reveal that AGR2 and GRP78 colocalise in both tissues and cell lines.

### AGR2 and GRP78 are highly expressed in glioblastoma CSCs

**Fig. 2:**
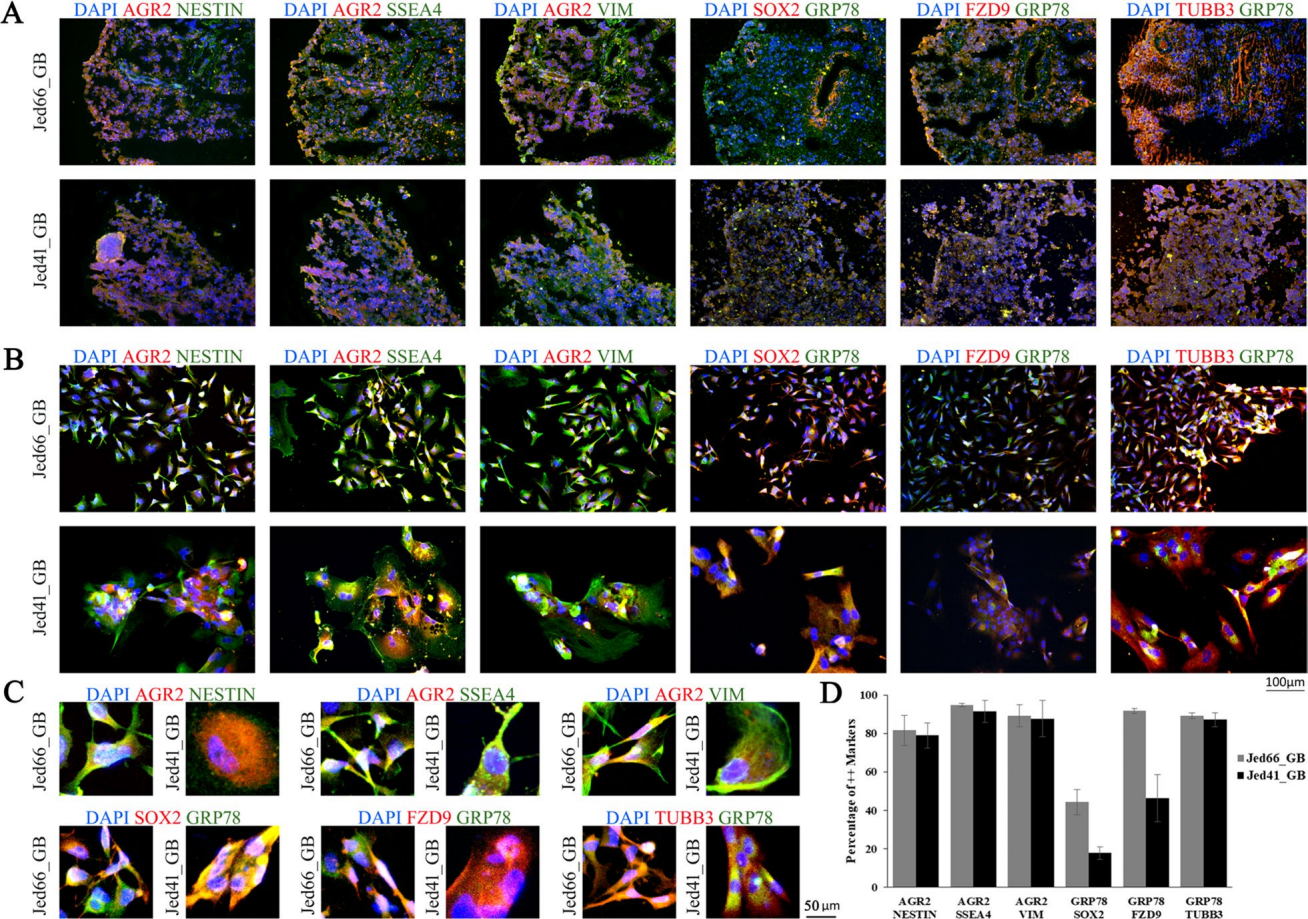
UPR markers co-staining with CSC markers. The immunofluorescence images for AGR2 (red) co-localising with either Nestin (green), SSEA4 (green), or VIM (green); and GRP78 (green) co-localising with either SOX2 (red), FZD9 (red), or TUBB3 (red) in **A**) fresh frozen tissues, **B**) corresponding primary cell lines and **C**) Magnified images to show detail intracellular localization of the respective proteins in the cells. DAPI is shown in blue. All images were taken at 20 × magnification. **D** A barograph showing the percentages of double-positive cells for the aforementioned markers in the primary cell lines. Error bars indicate the data variability between counts for three independent experiments

To investigate the expression of AGR2 and GRP78 in glioblastoma CSCs detected in tissues and corresponding cell lines, AGR2 was co-stained with Nestin, SSEA4 or Vimentin, and GRP78 was co-stained with SOX2, Frizzled 9 and TUBB3 (Fig. 2). All cells that expressed either AGR2 or GRP78 stained positive for several CSC markers. In both cell lines, Nestin, SSEA4, Vimentin, and TUBB3, were highly expressed (percentages ranged between 78.9 and 94.6%), while Frizzled 9 had a high/medium expression (an average of 91.7% in Jed66_GB and an average of 46.3% in Jed41_GB), and SOX2 had a medium/low expression (an average of 44.2% in Jed66_GB and an average of 17.7% in Jed41_GB). Additional markers, including CD133, GFAP and OLIG2, were also co-detected with the UPR proteins (Additional file 4: Figure S4).

### AGR2 remains expressed following drug treatment

**Fig. 3:**
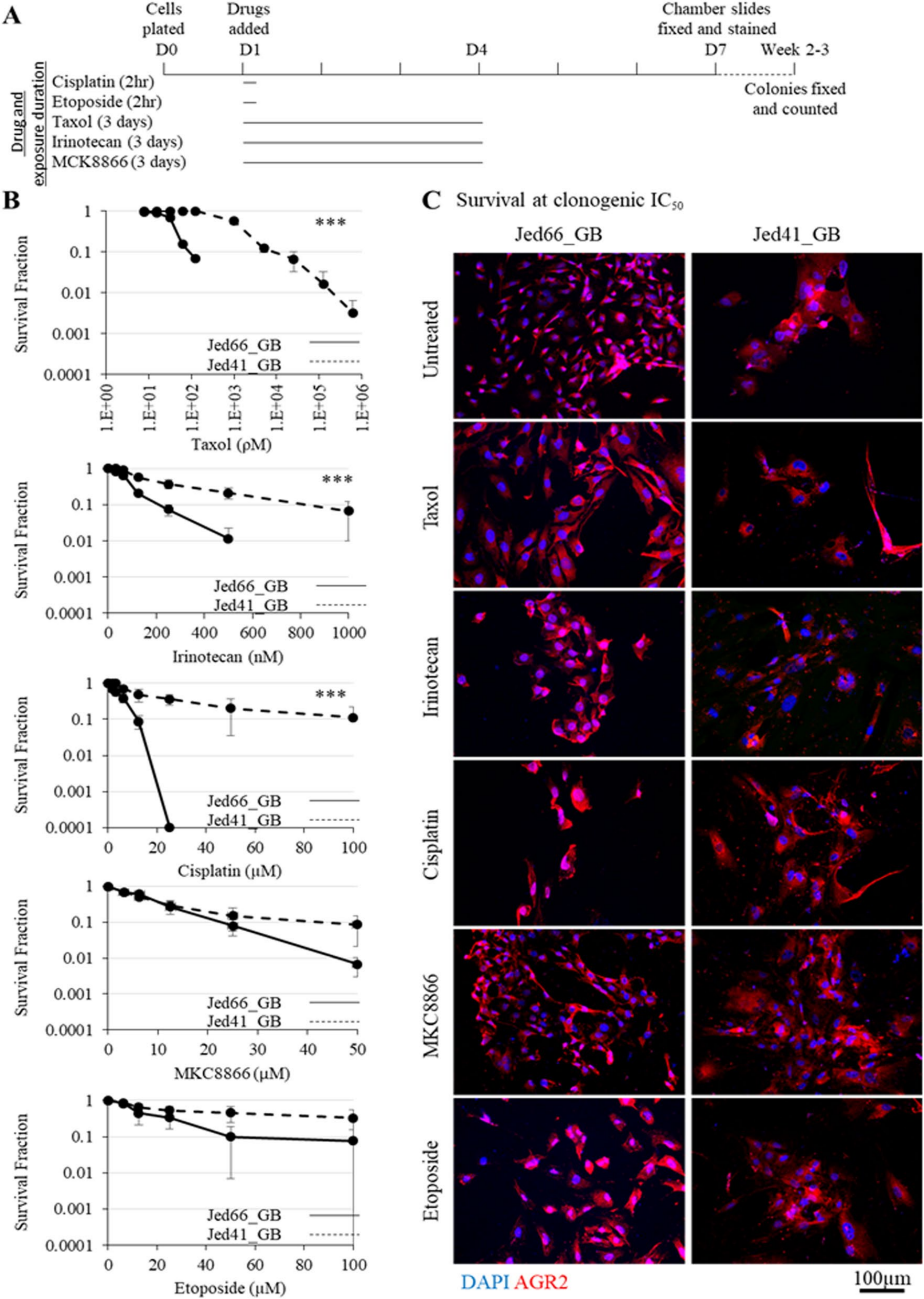
AGR2 expression in drug-exposed surviving cells. **A** A schematic diagram showing the timing of AGR2 detection following drug treatment. **B** Clonogenic growth evaluations of the primary cell lines treated with taxol, irinotecan, cisplatin, MKC8866 or etoposide. Error bars represent errors between counts for three independent experiments. Passage numbers for both cell lines ranged from 17 to 33. Three asterisks indicate significant difference at p < 0.001. **C** Immunofluorescence images for treated cells at IC_50_ values for the tested drugs. The Jed66_GB cell line was treated with taxol (42ρM), irinotecan (81 nM), cisplatin (4 μM), MKC8866 (7.4 μM) or etoposide (14 μM). The Jed41_GB cell line was treated with taxol (2 nM), irinotecan (210 nM), cisplatin (12 μM), MKC8866 (6.7 μM) or etoposide (29 μM). AGR2 is shown in red and DAPI in blue. All images were taken at 20 × magnification

To assess whether the expression of AGR2 remained high following drug treatment of Jed66_GB and Jed41_GB, both cell lines were drug-treated with a range of clinically applied drugs (Fig. 3). A clonogenic assay was used to observe the ability of cells to form clones following exposure to taxol, irinotecan, cisplatin, MKC8866 and etoposide. A comparison of the clonogenic IC_50_ revealed that both Jed66_GB and Jed41_GB showed high sensitivity to taxol and irinotecan (Taxol IC_50_ 42 ρM and 2 nM, respectively; irinotecan IC_50_ 81 nM and 210 nM, respectively). The sensitivity to cisplatin and MKC8866 for Jed66_GB and Jed41_GB were similar (cisplatin IC_50_ 4 μM and 12 μM, respectively; MKC8866 IC_50_ 7.4 μM and 6.7 μM, respectively). The least effective applied drug was etoposide (IC_50_ 14 µM for Jed66_GB and 29 μM for Jed41_GB). The overall responses to taxol, cisplatin or irinotecan were significantly higher in Jed66_GB than in Jed41_GB (Chiχ^2^ = 79.046, p < 0.001, Chiχ^2^ = 36.91, p < 0.001, Chiχ^2^ = 31.879, p < 0.001, respectively). Critically, the level of expression for AGR2 in both cell lines remained high in resistant surviving cells following the treatments with all drugs at IC_50_ concentrations (Fig. 3C).

### AGR2 exon 2B siRNA treatment repressed AGR2 protein expression and led to lower cell-densities in both cell lines

**Fig. 4:**
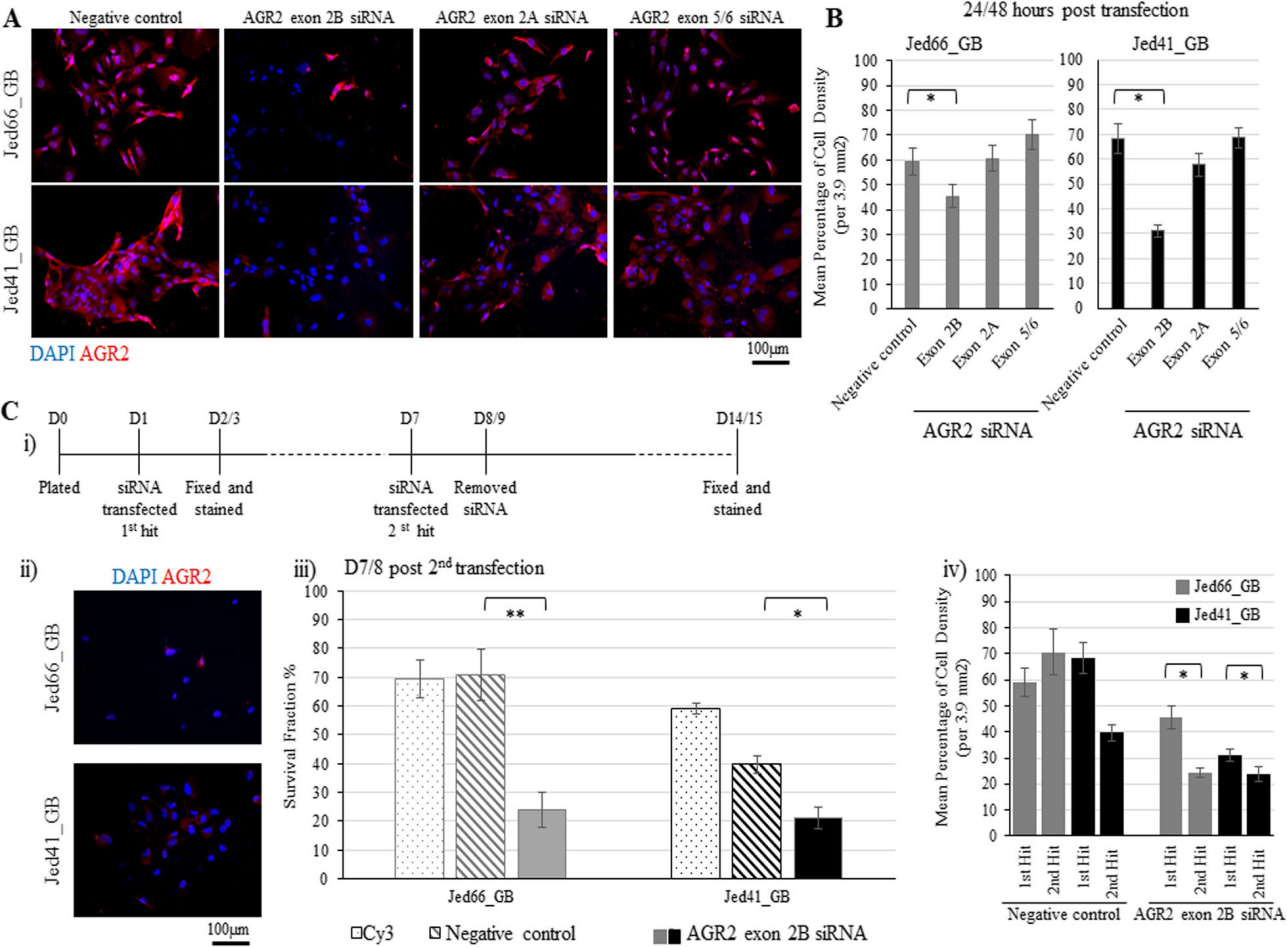
Repression of AGR2 in the primary cell lines. **A** Immunofluorescence images showing the expression of AGR2 following treatment using siRNA against AGR2 exons 2 and 5/6. AGR2 is shown in red and DAPI is shown in blue. All images were taken at 20x. **B** Barographs showing the mean cell densities within 3.9 mm^2^ for the primary cells transfected with the negative control or siRNA AGR2 oligos. Asterisks indicate significant Z-test differences at p < 0.05. Data were collected from three independent experiments. **C** Inhibition of AGR2 using a double-hit approach; i) A schematic diagram displaying the double-hit approach. ii) Immunofluorescence images showing the repression of AGR2 following two consecutive siRNA treatments using siRNA against AGR2 exon 2B. AGR2 is shown in red and DAPI in blue, and images were taken at 20 × magnification. iii) A barograph showing the survival fraction of cells transfected with Cy3, the negative control, or with two treatments of siRNA against AGR2 exon 2B. Two asterisks indicate significant T-test difference at p < 0.01. The error bars represent errors between counts for three independent experiments. iv) A barograph showing the mean cell densities within 3.9 mm^2^ for cells treated once or twice with negative control or siRNA against AGR2 exon 2B. The asterisk indicates a significant Z-test difference at p < 0.05. Data were collected from three independent experiments

To address whether the presence of AGR2 was essential for cell survival, RNAi-mediated AGR2 repression was applied to both cell lines. Cells were transfected with different concentrations of lipofectamine, and Cy3 and the negative control were used to optimise transfection rates (Additional file 5: Figure S5). Both cell lines were sensitive to the transfection reagents; thus, the maximum possible transfection rate that could be achieved while minimising cell death was approximately 50%. Three different regions of the gene were targeted with siRNA against exons 2 and 5/6. Clear inhibition of AGR2 expression was seen by immunofluorescence only when targeting exon 2B (Fig. 4A). Within 24/48 h of transfection, targeting exon 2B resulted in a significant reduction of the mean percentages of cell-densities compared with the controls (Fig. 4B). To investigate whether the remaining surviving cells following the first repression could be further AGR2-repressed, cells were treated with AGR2 exon 2B siRNA a second time, as illustrated in Fig. 4C i. Further repression of AGR2 was seen in Figure C ii, and cell densities were adversely affected in both cell lines compared to those exposed to the negative control and Cy3 (Fig. 4C iii). In addition, cell densities were significantly reduced compared with the first repression hit (Fig. 4C iv). However, at least 20% of the cells remained alive and were AGR2-expressed.

### Effects of AGR2 exon 2B siRNA treatment in combination with temozolomide or irinotecan

**Fig. 5:**
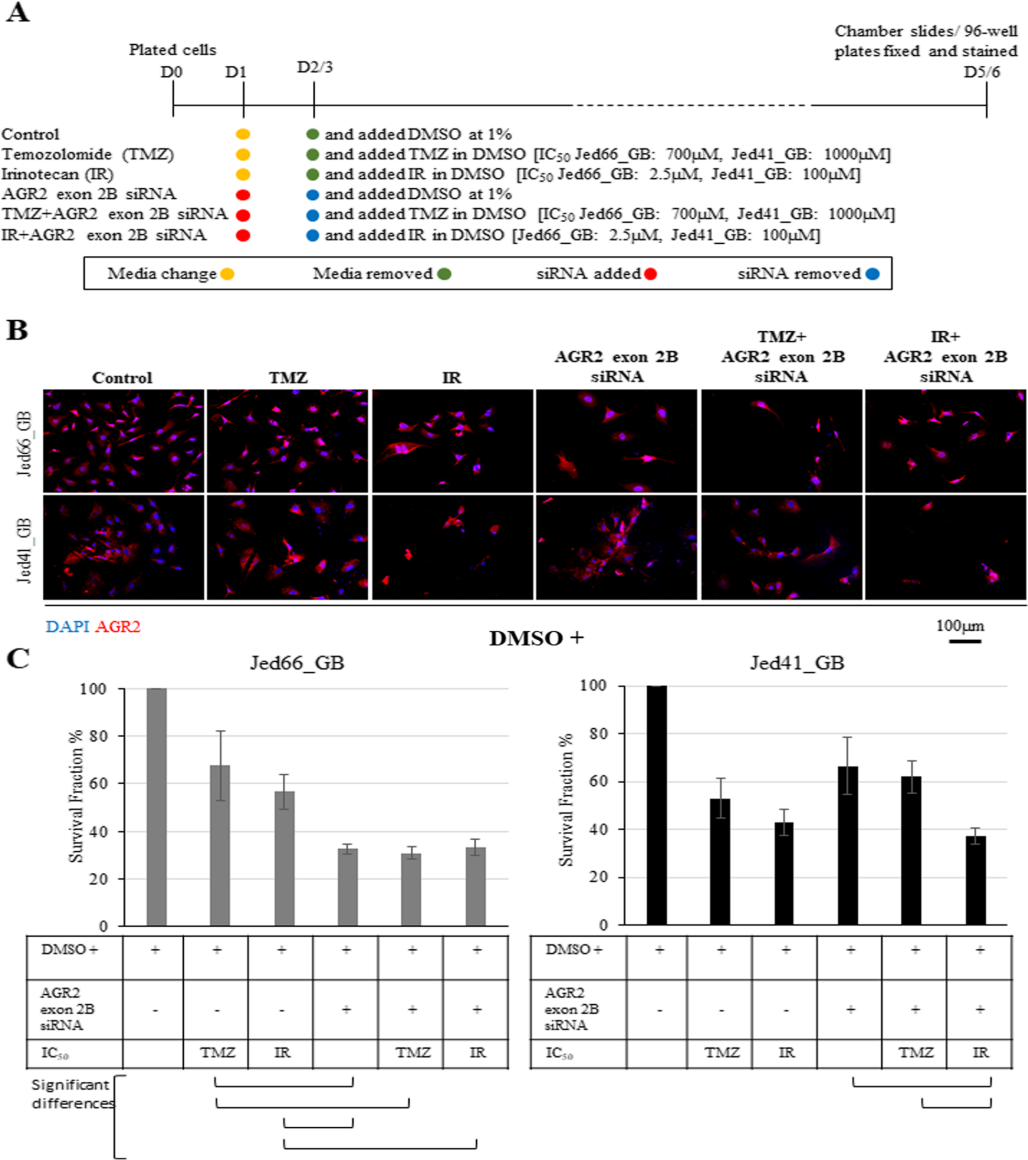
Inhibition of AGR2 exon 2B in conjunction with the clinically relevant drugs temozolomide (TMZ) and irinotecan (IR). **A** A schematic diagram showing the treatment protocols and the IC_50_ values. **B** Immunofluorescence images for treated cells at day 5/6. AGR2 is shown in red and DAPI in blue, and images were taken at 20x. **C** Barographs displaying the survival fractions of the primary cell lines following individual and co-treatments using siRNA against AGR2 exon 2B with temozolomide or with irinotecan. The error bars represent errors between counts for three independent experiments. The connective lines indicate a significant T-test difference at p < 0.05

To address the effects of AGR2 exon 2B siRNA treatment in conjunction with the clinically relevant drugs temozolomide and irinotecan, the survival fraction of both cell lines was assessed following single or combination treatments of growth inhibition and siRNA (Fig. 5). For Jed66_GB, AGR2 exon 2B siRNA treatment alone was significantly more effective than single treatment with either drug (AGR2 exon 2B siRNA versus temozolomide, T-test p < 0.05, AGR2 exon 2B siRNA versus irinotecan, T-test p < 0.05). Exposure to either drug in combination with siRNA treatment had similar effects when using only siRNA treatment. For Jed41_GB, compared to the control, AGR2 exon 2B siRNA treatment was as effective as a single treatment with either drug. Exposure to irinotecan in combination with siRNA treatment was the most effective treatment and was significantly more effective than using temozolomide with siRNA (T-test: p < 0.05).

### Early repression of AGR2 has cell line-dependent effects

**Fig. 6:**
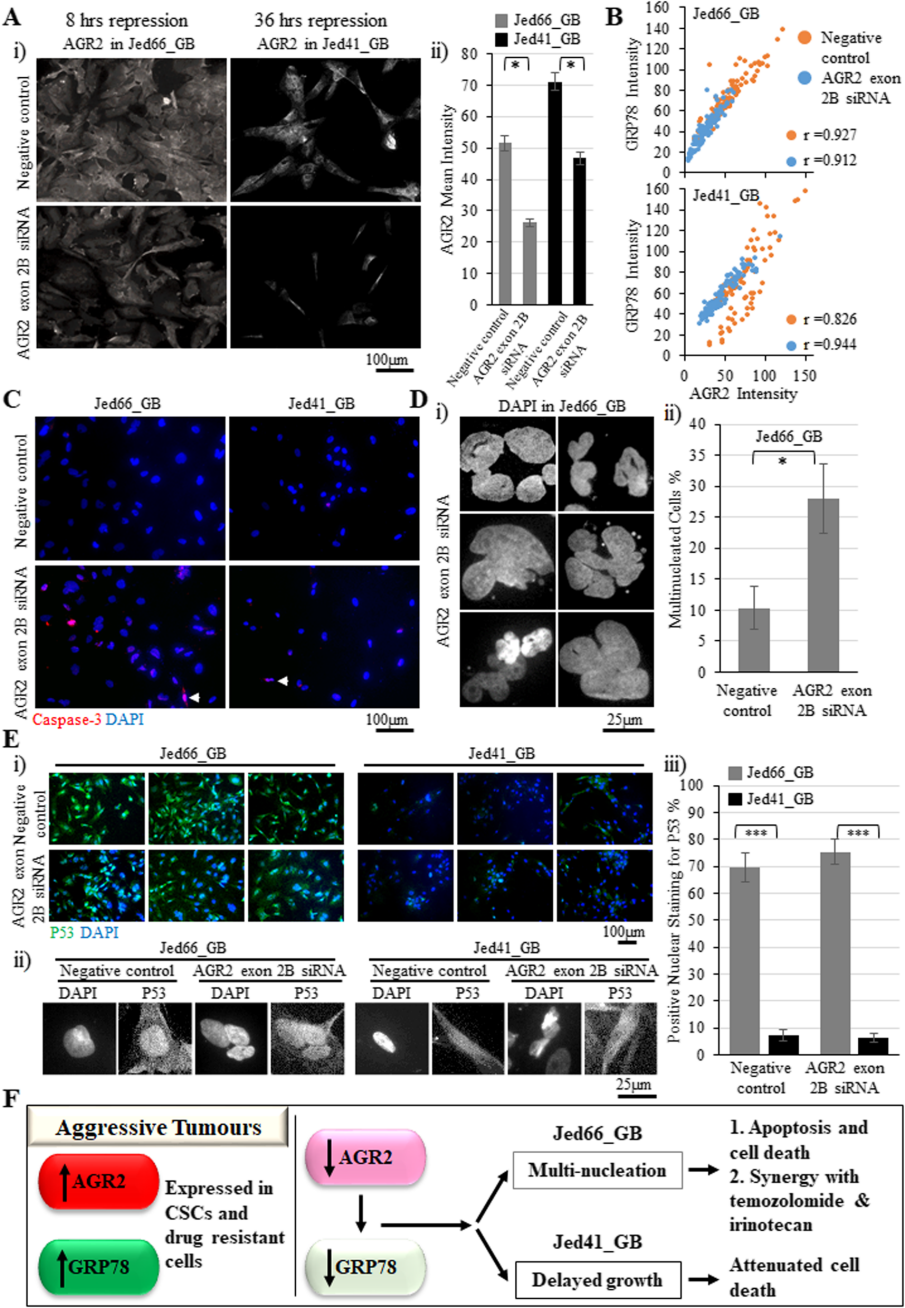
The effects of early repression of AGR2 in both Jed66_GB and Jed41_GB. **A** i) Images showing minor repression of AGR2 in both cell lines following the short post-transfection of eight hours for Jed66_GB and 36 h for Jed41_GB. ii) The AGR2 mean intensities for negative control and AGR2 exon 2B siRNA-transfected cells. The asterisk indicates significant T-test difference at p < 0.05. **B** GRP78 expression and AGR2 expression in both negative control and AGR2 exon 2B siRNA-transfected cells for both cell lines. **C** Immunofluorescence images of nuclear Caspase-3 in negative control and AGR2 exon 2B siRNA-transfected cells. The arrow points to a Caspase-3-positive multinucleated cell. Caspase-3 is shown in red and DAPI in blue. **D** i) Images show multinucleation in Jed66_GB ii) The percentage of multinucleated cells in Jed66_GB following negative control and AGR2 exon 2B siRNA transfections. Asterisk indicate T-test significant difference at p < 0.05. **E** i) Immunofluorescence images displaying the expression of P53 in both cell lines following negative control and AGR2 exon 2B siRNA transfections. P53 is shown in green and DAPI in blue. ii) Magnified images showing P53 localisation. iii) The percentage of cells positive for nuclear P53 was significantly higher in Jed66_GB compared with Jed41_GB. Three asterisks indicate T-test significant difference at p < 0.001. **F** A suggested model that explains the role of AGR2 in glioblastoma. Both in situ and in vitro, AGR2 and GRP78 are highly expressed in CSCs and drug-resistant cells. Upon repression of AGR2, GRP78 was also reduced. Early cell fate seems to be dependent on the used model, and can lead either to multinucleation followed by cell death or to delayed growth followed by attenuated cell death

To understand how AGR2 repression may result in the reduction of cell densities or cell death, cells were treated with either negative control siRNA or AGR2 exon 2B siRNA and then analysed following the shortest possible post-transfection periods determined for both cell lines (Fig. 6). A minor reduction of AGR2 expression was detected in Jed66_GB following eight hours of incubation after transfection, and, in Jed41_GB, following 36 h of incubation after transfection (Fig. 6A). An analysis of single-cell intensities indicated a strong correlation between the expression of AGR2 and GRP78 in control and AGR2 exon 2B siRNA-treated cells, and a reduction of GRP78 expression was seen upon repressing AGR2 (Fig. 6B). In addition, Caspase-3 staining indicated that nuclear expression was significantly increased in both cell lines following AGR2 exon 2B siRNA transfection compared to the negative control siRNA transfection (percentages of Caspase-3 positive cells, Jed66_GB negative control: 9.48 ± 3.29%, AGR2 exon 2B: 38.35 ± 6.03%, T-test p < 0.01; Jed41_GB negative control: 2.06 ± 0.63%, AGR2 exon 2B: 6.89 ± 2.6%, T-test: p < 0.05), (Fig. 6C). However, Jed66_GB had a significantly higher nuclear Caspase-3 staining than Jed41_GB in both the negative control siRNA and AGR2 exon 2B siRNA transfected cells (T-test: p < 0.05 and T-test: p < 0.01, respectively). Critically, the slight repression of AGR2 resulted in the appearance of multinucleation in Jed66_GB, but not in Jed41_GB (Fig. 6D).

Since we detected the presence of damaging variants in *P53* in Jed41_GB but not in Jed66_GB, and since P53 has a critical role in driving apoptosis, we investigated the presence of nuclear P53 in glioblastoma cells. The presence of nuclear P53 was significantly higher in Jed66_GB compared with Jed41_GB in both the negative control siRNA and AGR2 exon 2B siRNA transfected cells (p < 0.001) (Fig. 6E).

## Discussion

This study focused on understanding the importance of the metastatic-related biomarker AGR2 and the UPR protein GRP78 in glioblastoma tissues and corresponding primary cell lines, with particular attention paid to CSCs and drug resistance. We determined the levels of AGR2 protein expression in recurrent glioblastoma tissues and primary cell lines and showed its co-expression with GRP78 and several CSC markers. We investigated the relationship between AGR2 expression and drug resistance for classically known chemotherapeutics and assessed the effects on cell survival following the repression of AGR2 using siRNA, in combination with chemotherapeutic drugs. In addition, we analysed the early effects of repressing AGR2 and proposed a likely mode of action (Fig. 6F).

Some studies have previously analysed the role of AGR2 or GRP78 in standard glioblastoma cell lines [31, 41]. In our research, we utilised two primary glioblastoma cell lines, adult Jed66_GB and paediatric Jed41_GB, as primary cell lines represent the ‘central dogma’ of patients’ tumours more accurately than commercially available cell lines [42]. The limitation of using such cell lines is the availability of primary cell lines that are highly adapted to in vitro conditions, can be maintained at relatively low passages and can be harvested for experimentations in sufficient cell numbers. Both Jed66_GB and Jed41_GB were able to grow fast to enable investigations to be carried out in cell passages below 35. Both cell lines were able to form clones and had a high percentage of Ki67- and BMI-positive cells. In addition, both cell lines indicated a high degree of migration in the scratch assay. A whole exome sequencing for Jed66_GB indicated the presence of TC-rare damaging COSMIC variants detected in genes that were previously associated with glioblastoma including BCR activator of RhoGEF and GTPase (*BCR*) [43, 44], TNF receptor associated protein 1 (*TRAP1*) [45–47], DNA polymerase delta 1, catalytic subunit (*POLD1*) [48], otopetrin 1 (*OTOP1*) [49], tyrosine kinase 2 (*TYK2*) [50], AT-rich interaction domain 1B (*ARID1B*) [51], CD48 molecule (*CD48*) [52], ubiquitin specific peptidase 18 (*USP18*) [53], nuclear receptor corepressor 1 (*NCOR1*) [54], NFE2 Like BZIP Transcription Factor 2 (*NFE2L2*) [55], and Kinesin Family Member 1A (*KIF1A*) [56]. For Jed41_GB, exome sequencing showed the presence of TC-rare damaging COSMIC variants detected in glioblastoma-associated genes including *TP53* [43, 44, 57], LDL receptor related protein 1B (*LRP1B*) [44, 58], adhesion G protein-coupled receptor E5 (*ADGRE5*) [59], atrophin 1 (*ATN1*) [60], autophagy related 2B (*ATG2B*) [61, 62], MYC associated zinc finger protein (*MAZ*) [23, 63], WNK lysine deficient protein kinase 1 (*WNK1*) [64, 65], UDP glucuronosyltransferase family 1 member A1 (*UGT1A1*) [66], and UDP glucuronosyltransferase family 1 member A6 (*UGT1A6*) [67]. Not surprisingly, differences in functional characteristics between the two cell lines were demonstrated, consistent with the high inter-patient heterogeneity seen for glioblastoma [6–9, 15].

Immunofluorescence results showed, for the first time, a clear subcellular co-localisation for AGR2 with GRP78 in glioblastoma in situ and in the corresponding cell lines. This is consistent with the notion that AGR2 is an interacting partner of GRP78 [17, 24]. In addition, both proteins had an elevated expression, a phenomenon previously detected in glioblastoma [19, 32, 68]. Notably, AGR2 is not expressed in normal brain tissues [26], and it was shown to be preferentially expressed in high grade meningiomas compared to low grade meningiomas [39]. AGR2 overexpression has been linked to higher ER stress [69, 70], possibly via several modulators [17, 32]. Surprisingly, AGR2 was detected in the nucleus in both cell lines, a unique feature that was not seen in situ, suggesting that AGR2 behaviour is likely to be responsive to the tumour microenvironment [71, 72].

CSCs are well identified in glioblastoma [7, 9], and several associated markers have been studied [13, 14]. The finding that both AGR2 and GRP78 proteins are colocalised with CSC markers in the same tissues as well as in the corresponding cell lines suggests a functional interaction of AGR2 and GRP78 in glioblastoma-CSCs. Several studies have shown an indirect correlation between the expression of either protein and CSCs markers [20, 31]. The high expression of AGR2 has been previously associated with the expression of Nestin, CD133 and SOX2 in high-grade meningioma tissues [39]. AGR2 expression was also shown to have a strong positive correlation with nanog homeobox (NANOG) in oral squamous cell carcinoma [73]. The effect of high GRP78 expression in glioblastoma-CSCs is still being investigated. A previous study demonstrated that using the ER stressor drug thapsigargin resulted in the increased expression of GRP78, and the reduced expression of SOX2 in glioblastoma patient derived CSCs [20]. However, in that work, SOX2 downregulation was not shown to be directly affected by the UPR pathway. The data presented here show the co-presence of GRP78 in cells expressing SOX2, FZD9, OLIG2, GFAP or TUBB3, suggesting that the presence of GRP78 does not directly inhibit the aforementioned CSC markers. In addition, similar to other studies, Ki67 expression was seen in a few cells that express GRP78 [74, 75], indicating that proliferation is not inhibited in UPR-active CSCs.

The expression of AGR2 remained high in drug-resistant cells following treatments with a range of clinically applied drugs that utilize different modes of action [76–80]. This suggests that the presence of AGR2 is critical for glioblastoma cell survival, regardless of drug type. Interestingly, of all the applied drugs, the primary cell lines were most sensitive to taxol, an observation previously noted in glioblastoma-standard cell lines [81, 82]. The endoplasmic reticulum to nucleus signalling 1 (ERN1), otherwise known as bifunctional endoribonuclease/protein kinase (IRE1), is a UPR activator and has been suggested as an influential regulator of AGR2 [17]. The ERN1 inhibitor, MKC8866, was less effective in inhibiting clone formations than irinotecan or taxol; however MKC8866 was similarly effective in both cell lines, suggesting that the UPR machinery is equally active in the studied cell lines.

Short and prolonged RNAi-mediated AGR2 exon 2B repression led to lower cell densities in both cell lines. Exon 2 covers nucleotides 66–211 and its transcript is thought to be present in the AGR2 full lengths, AGR2vC and AGR2vH isoforms [25]. In this study, two siRNAs were used to target exon 2; however, only AGR2 exon 2B siRNA, effecting a region that codes for amino acids 33–40 AA (TKDSRPKL), was detrimental. Previously, repression of AGR2 via targeting exon 7 or exon 8 in glioblastoma standard cell lines showed disruptions in the cell cycle, cell growth, migration, and cell invasion [41, 83, 84]. In colorectal cancer cells, AGR2 exon 2 knockout was shown to be crucial for protein-mediated cell adhesion, and resulted in the increase of reaction oxygen species production [85]. This suggests that targeting different regions of AGR2 results in different responses that may also be cell line dependent.

Knocking down AGR2 using siRNA against AGR2 exon 2B in the primary cell lines had partially synergistic effects with the glioblastoma-associated drugs temozolomide and irinotecan. AGR2 knockout, in combination with other drugs, was previously shown to be effective in inhibiting the growth of different cell line models [85–88]. To the best of our knowledge, this is the first study to show observations related to the effect of siRNA against AGR2 exon 2 in combination with either temozolomide or irinotecan. Inhibiting AGR2 exon 2B was significantly more effective than a single treatment with either drug in Jed66_GB, and exposure to either drug with siRNA did not affect the survival fraction that was achieved when using siRNA only. However, this was not necessarily the case for Jed41_GB. This is consistent with the notion that heterogeneity in glioblastoma patients has an impact on drug response [8, 9]. Thus, although inhibiting AGR2 exon 2B may be therapeutically effective, a preselection of sensitive tumours is likely to be necessary. Further work is needed to decipher the exact mechanism of action for the interplay between AGR2 inhibition and the aforementioned drugs.

How the repression of AGR2 could lead to the reduction of cell densities or cell death was partially clarified when observations for the earliest time points of repression were made for Jed66_GB and Jed41_GB (8 h and 36 h, respectively). Both cell lines increased nuclear Caspase-3 activation following a slight reduction in AGR2, a feature seen previously in primary breast cancer cells [87]. However, the early effects seen following repression were different between the two cell lines. First, Jed66_GB had a higher capacity to activate nuclear Caspase-3 than Jed41_GB in the controls and repressed cells. This is perhaps due to the presence of wild-type *P53* in Jed66_GB compared with mutated *P53* in Jed41_GB. This is also consistent with the observations that Jed41_GB is more resistant to taxol, cisplatin and irinotecan compared with Jed66_GB. However, the higher capacity of Jed66_GB to proliferate could contribute to better overall recovery following treatment.

Second, early repression of AGR2 led to multinucleation only in Jed66_GB. This indicates that the pathway to induce apoptosis following AGR2 repression is cell line-dependent. How can the repression of AGR2 lead to apoptosis via multiple pathways? It was previously known that AGR2 can affect the presence of multiple molecules [17, 86, 89, 90]. This global impact of AGR2 is likely to occur via modulating the ER degradation enhancing, alpha-mannosidase-like protein 1 (EDEM), which is a critical component in the ERAD system [17, 83]. One can postulate that in a system similar to Jed66_GB, where proliferation is high, and defects in genes such as *PLOD1* that are associated with nuclear integrity exist [91], the repression of AGR2 could then interfere with the cytoskeleton, via increasing the degradation of β-dystroglycan [92], which is a basement membrane component required to bind to actin and is critical for the integrity of the cytoskeleton [93]. Alternatively, in a cell line with wild-type *p53*, where the threshold of nuclear integrity is dependent on a functional P53, repression of AGR2 may interfere with P53, as previously shown, and cause multinucleation [94, 95]. In contrast, in a system such as Jed41_GB, where cells grow slower, have adapted to dysfunctional genes that are important for nuclear integrity such as *P53*, leukaemia NUP98 fusion partner 1 (*LNP1*) and Sad1 and UNC84 domain containing 2 (*SUN2*) [96, 97], the repression of AGR2 could then directly induce apoptosis by enabling the degradation of the anti-apoptotic proteins B-cell lymphoma 2 (Bcl2) and BCL2 Like 1(Bcl2l1), as previously seen in head and neck squamous cell carcinoma cell lines [98].

## Conclusion

In conclusion, the data demonstrate that AGR2 and GRP78 are highly expressed in glioblastoma CSCs and drug-resistant cells in situ and in vitro. The repression of AGR2 exon 2B using siRNA resulted in the reduction of cell densities and was synergistically effective in decreasing cell densities of temozolomide- and irinotecan-treated Jed66_GB cells. The suppression of AGR2 influenced the presence of GRP78, possibly through navigating the UPR machinery. Early cell fate seemed to depend on the presence of wild-type P53 and can either lead to multinucleation followed by cell death or to delayed growth followed by attenuated cell death. Further work is critical to investigate the relationship between AGR2 and P53 and their impact on cell death following drug treatment in a larger cohort.

## Supplementary Information

**Additional file 1: Figure S1.** Histopathological classifications. Jed66_GB has malignant glial cells palisade around a central focus of necrosis and microvascular proliferations on the left and right sides (H&E stain; power 200 X). The tumour is positive for GFAP immunostaining (Power 100 X). Jed41_GB has numerous large bizarre multinucleated tumour cells on the right side and tumour necrosis on the left side (H&E stain; power 400 X). The tissue was GFAP positive (Power 100 X).

**Additional file 2: Figure S2.** Biological characteristics of the glioblastoma primary cell lines Jed66_GB and Jed41_GB. A**) **Immunofluorescence images showing i) the expression of AGR2, ii) Nuclear localisation of AGR2. AGR2 is shown in red and DAPI in blue. Images were taken at 20x. B) A barograph displaying the doubling time for each cell line. Error bars represent errors between counts for three independent experiments. C) A line graph showing the clonogenic capacity for Jed66_GB cells (Passages 17-23) and Jed41_GB cells (Passages 8–14). Error bars represent errors between counts for three independent experiments. D) Immunofluorescence images showing the expression of Ki67 (red) or BMI (green) in both primary cell lines. Images were taken at 20x. E) A barograph showing the average percentage of cells positive for Ki67 or BMI. The error bars represent errors between counts for three independent experiments. F) Images of cells that underwent a scratch assay taken on day 0 before and after scratch and at days 3 and 4 after the gap was filled. Images were taken at 5x magnification.

**Additional file 3: Figure S3.** Sequencing variants in the primary cell lines and corresponding tissues. A) Conventional sequencing for two critically damaging variants present in either cell line with its corresponding tissue, *STC2* in Jed66_GB and *TP53* in Jed41_GB. B) Only non-damaging and common variants for AGR2 or GRP78 were detected in either cell line. C) Prominent functions likely to be affected for both cell lines, as per related TC rare damaging COSMIC variants. Prominent functions are coloured in grey, genes affected in Jed66_GB are coloured in blue and those identified in Jed41_GB are coloured in green.

**Additional file 4: Figure S4.** Further UPR markers co-stained with CSC markers in the primary cell lines. A) Immunofluorescence images for AGR2 (red) co-stained with CD133 (green), and GRP78 (green) co-stained with GFAP (red), or with OLIG2 (Red). DAPI is shown in blue. Images were taken at 20x magnification. B) Magnified images to show detail intracellular localization of the respective proteins. C) A barograph showing the percentages of double-positive cells for the aforementioned markers in the primary cell lines. Error bars represent errors between counts for three independent experiments.

**Additional file 5: Figure S5.** siRNA optimisation for the primary cell lines. A) Barographs showing the survival fractions for the Cy3 transfection control and siRNA negative control at optimum concentrations of lipofectamine. Error bars represent errors between counts for three independent experiments. B) Immunofluorescence images showing the Cy3-positive cells are shown in red, and DAPI is shown in blue. Images were taken at 20x magnification. C) A barograph showing the percentages of Cy3-positive cells. Error bars represent errors between counts for three independent experiments. D) Immunofluorescence images showing cell densities following transfections, and the images were taken at 5x magnification. Cy3-positive cells are shown in red, and DAPI is shown in blue.

**Additional file 6: Table S1.** Rare damaging COSMIC variants detected in the tissue and cell lines for Jed66_GB.

**Additional file 7: Table S2.** Rare damaging COSMIC variants detected in the tissue and cell lines for Jed41_GB.

**Additional file 8: Table S3.** It is likely affect prominent functions in the studied cell lines. Genes with rare damaging COSMIC variants were detected in the tissue and corresponding cell line for each patient and organized by prominent functions (Panther: Molecular Function/ Biological Process/Pathway/Reactome Pathway, NCBI Gene information*) for both Jed66_GB and Jed41_GB. Only variants with a possible/probable damaging PolyPhen effect were included, as per data annotated by BaseSpace or as detected manually using PolyPhen-2 Wiki.

## Data Availability

The datasets used and/or analysed during the current study are available from the corresponding author on reasonable request.

## Abbreviations

ADGRE5: Adhesion G protein-coupled receptor E5
AGR2: Anterior gradient homologue 2
AKT3: AKT serine/threonine kinase 3
ARID1B: AT-rich interaction domain 1B
ATG2B: Autophagy related 2B
ATN1: Atrophin 1
Bcl2: B-cell lymphoma 2
Bcl2l1: BCL2 Like 1
BCR: BCR activator of RhoGEF and GTPase
BMI1: BMI1 proto-oncogene, polycomb ring finger
CD133: Prominin 1
CD48: CD48 molecule
CDKN2A: Cyclin dependent kinase inhibitor 2A
CNSTs: Central nervous system tumours
COSMIC: Catalogue of somatic mutations in cancer
CSCs: Cancer stem cells
CTNNB1: Catenin beta 1
DT: Doubling time
EDEM: Enhancing alpha-mannosidase like protein 1
EGFR: Epidermal growth factor receptor
ER: Endoplasmic reticulum
ERAD: Endoplasmic-reticulum-associated protein degradation
ERN1: Endoplasmic reticulum to nucleus signalling 1
FGFR2: Fibroblast growth factor receptor 2
FZD9: Frizzled class receptor 9
GB: Glioblastoma
GFAP: Glial fibrillary acidic protein
GRP78: Glucose regulatory protein 78/BIP
IRE1: Endoribonuclease/protein kinase
IRS2: Insulin receptor substrate 2
IDH1/2: Isocitrate dehydrogenase (IDH) 1 and 2, KIF1A: kinesin family member 1A
LNP1: Leukaemia NUP98 fusion partner 1
LRP1B: LDL receptor related protein 1B
MAZ: MYC associated zinc finger protein
NANOG: Nanog homeobox
NCOR1: Nuclear receptor corepressor 1
NF1: Neurofibromin 1
NFE2L2: NFE2 Like BZIP transcription factor 2
NOTCH1: Notch receptor 1
NUDCD3: Nudc domain containing 3
OLIG2: Oligodendrocyte transcription factor 2
OTOP1: Otopetrin 1
P53: Tumour protein p53
Park2: Parkin RBR E3 ubiquitin protein ligase
PFA: Paraformaldehyde
POLD1: DNA polymerase delta 1, catalytic subunit
PTEN: Phosphatase and tensin homolog
PTPred: Protein tyrosine phosphatase receptor type D
siRNA: Small interfering duplex RNA sequences
SOX2: SRY-Box transcription factor 2
SSEA4: Stage-specific embryonic antigen-4
STC2: Stanniocalcin-2
SUN2: Sad1 and UNC84 domain containing 2
TC: Tissue cell line
TM: Transfection media
TRAP1: TNF receptor associated protein 1
TUBB3: Tubulin beta 3 class III
TYK2: Tyrosine kinase 2
UGT1A1: UDP glucuronosyltransferase family 1 member A1
UGT1A6: UDP glucuronosyltransferase family 1 member A6
UPR: Unfolded protein response
USP18: Ubiquitin specific peptidase 18
VCF: Variant call format
VIM: Vimentin
WES: Whole exome sequencing
WNK1: WNK lysine deficient protein kinase 1
