# Supplementary figures and images for: The anterior gradient homologue 2 (AGR2) co-localises with the glucose-regulated protein 78 (GRP78) in cancer stem cells, and is critical for the survival and drug resistance of recurrent glioblastoma: in situ and in vitro analyses
Source: Cancer Cell Int. 2022 Dec 8;22:387. doi: 10.1186/s12935-022-02814-5 (PMC9730595; doi:10.1186/s12935-022-02814-5)

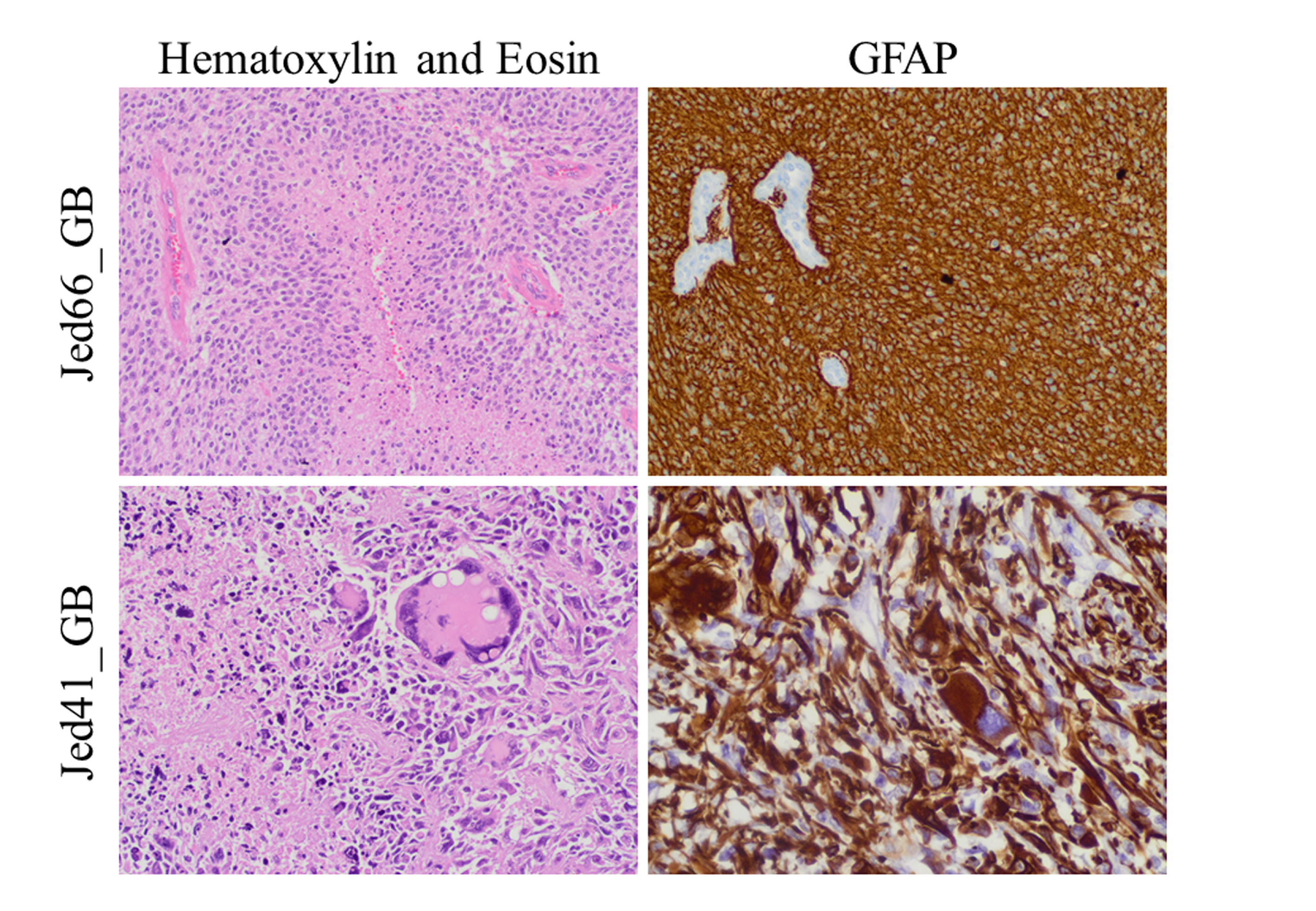

Supplement: Supplementary file 1 — Additional file 1: Figure S1. Histopathological classifications. Jed66_GB has malignant glial cells palisade around a central focus of necrosis and microvascular proliferations on the left and right sides (H&E stain; power 200 X). The tumour is positive for GFAP immunostaining (Power 100 X). Jed41_GB has numerous large bizarre multinucleated tumour cells on the right side and tumour necrosis on the left side (H&E stain; power 400 X). The tissue was GFAP positive (Power 100 X). [file 12935_2022_2814_MOESM1_ESM.tif]

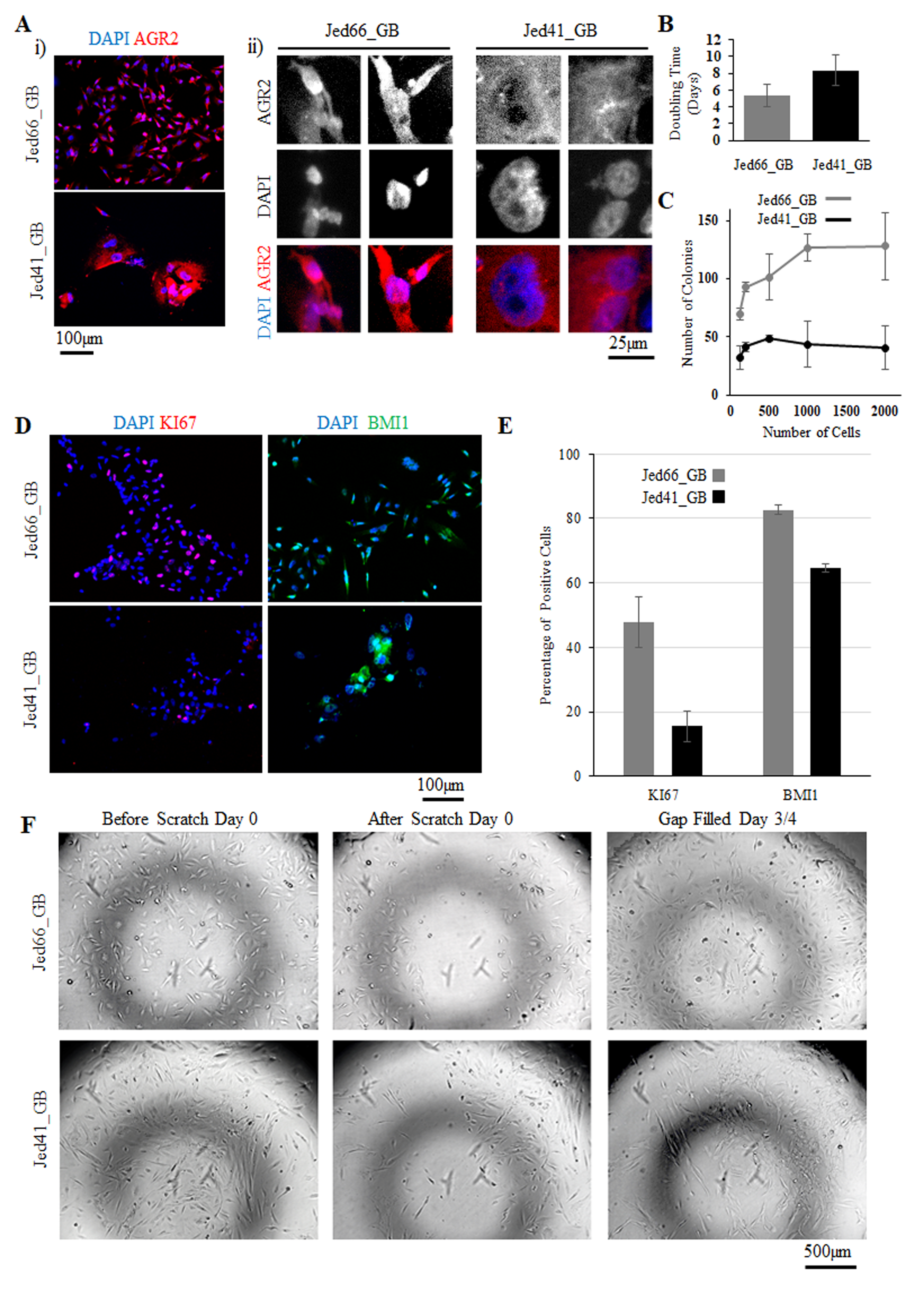

Supplement: Supplementary file 2 — Additional file 2: Figure S2. Biological characteristics of the glioblastoma primary cell lines Jed66_GB and Jed41_GB. A) Immunofluorescence images showing i) the expression of AGR2, ii) Nuclear localisation of AGR2. AGR2 is shown in red and DAPI in blue. Images were taken at 20x. B) A barograph displaying the doubling time for each cell line. Error bars represent errors between counts for three independent experiments. C) A line graph showing the clonogenic capacity for Jed66_GB cells (Passages 17-23) and Jed41_GB cells (Passages 8–14). Error bars represent errors between counts for three independent experiments. D) Immunofluorescence images showing the expression of Ki67 (red) or BMI (green) in both primary cell lines. Images were taken at 20x. E) A barograph showing the average percentage of cells positive for Ki67 or BMI. The error bars represent errors between counts for three independent experiments. F) Images of cells that underwent a scratch assay taken on day 0 before and after scratch and at days 3 and 4 after the gap was filled. Images were taken at 5x magnification. [file 12935_2022_2814_MOESM2_ESM.tif]

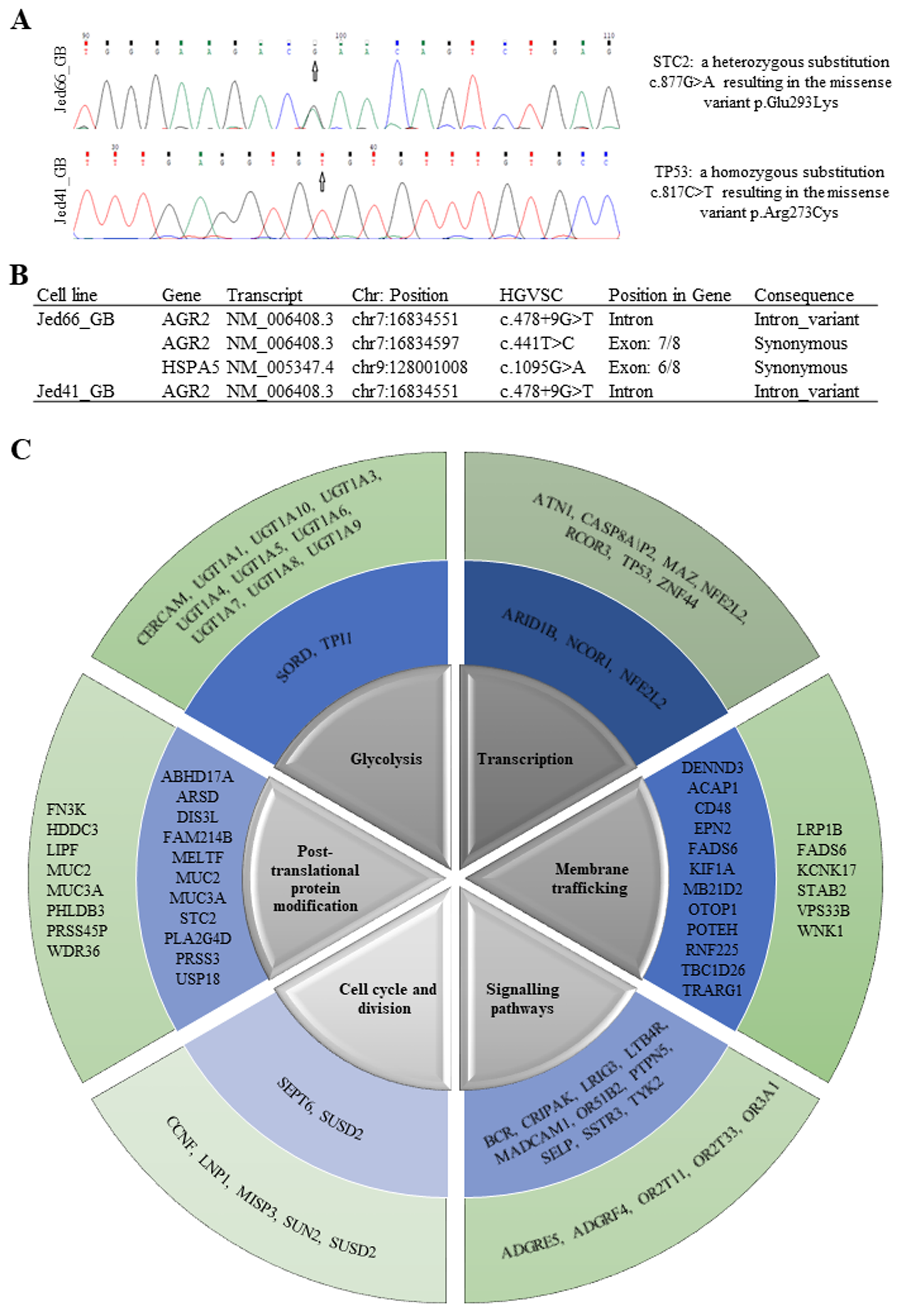

Supplement: Supplementary file 3 — Additional file 3: Figure S3. Sequencing variants in the primary cell lines and corresponding tissues. A) Conventional sequencing for two critically damaging variants present in either cell line with its corresponding tissue, STC2 in Jed66_GB and TP53 in Jed41_GB. B) Only non-damaging and common variants for AGR2 or GRP78 were detected in either cell line. C) Prominent functions likely to be affected for both cell lines, as per related TC rare damaging COSMIC variants. Prominent functions are coloured in grey, genes affected in Jed66_GB are coloured in blue and those identified in Jed41_GB are coloured in green. [file 12935_2022_2814_MOESM3_ESM.tif]

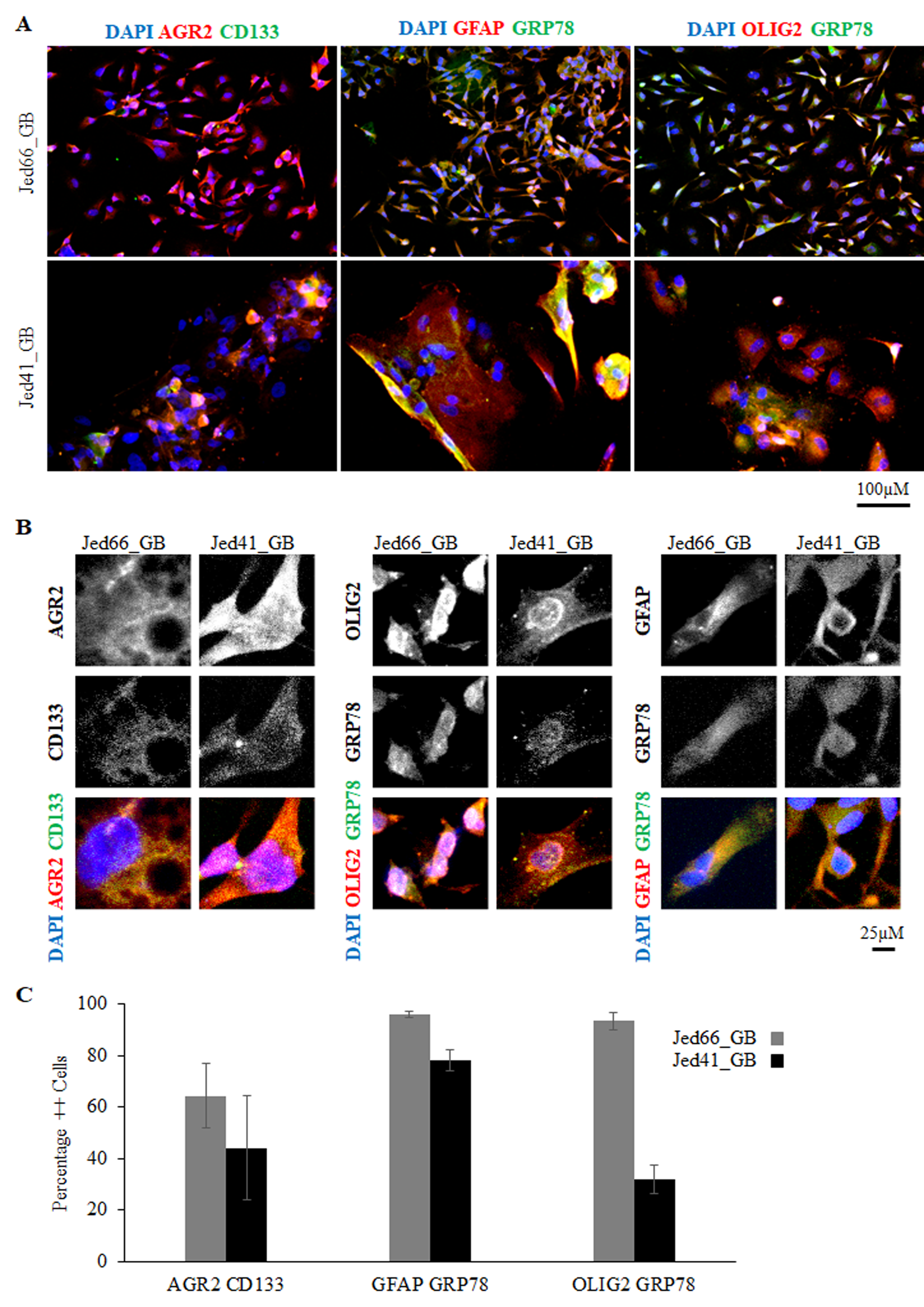

Supplement: Supplementary file 4 — Additional file 4: Figure S4. Further UPR markers co-stained with CSC markers in the primary cell lines. A) Immunofluorescence images for AGR2 (red) co-stained with CD133 (green), and GRP78 (green) co-stained with GFAP (red), or with OLIG2 (Red). DAPI is shown in blue. Images were taken at 20x magnification. B) Magnified images to show detail intracellular localization of the respective proteins. C) A barograph showing the percentages of double-positive cells for the aforementioned markers in the primary cell lines. Error bars represent errors between counts for three independent experiments. [file 12935_2022_2814_MOESM4_ESM.tif]

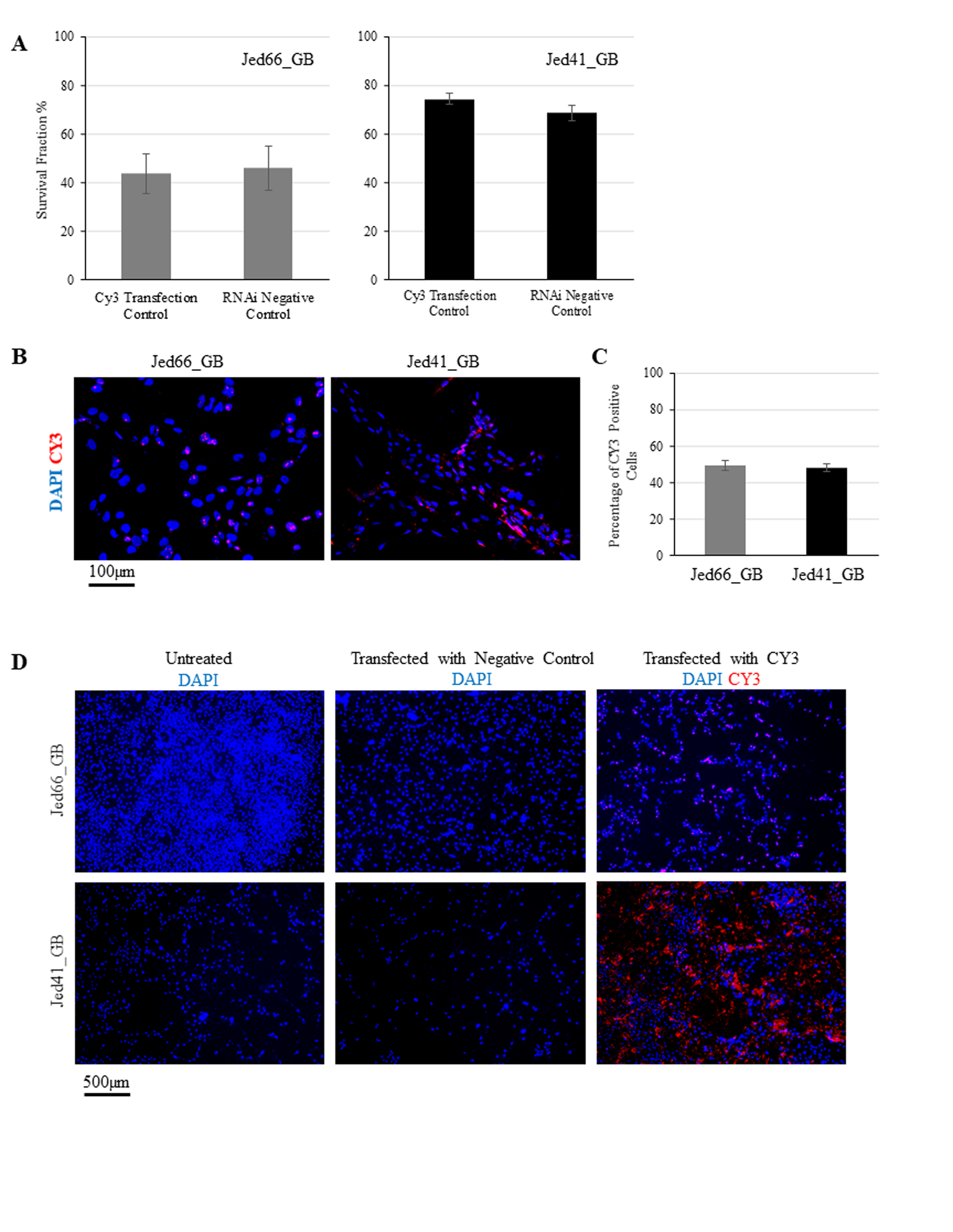

Supplement: Supplementary file 5 — Additional file 5: Figure S5. siRNA optimisation for the primary cell lines. A) Barographs showing the survival fractions for the Cy3 transfection control and siRNA negative control at optimum concentrations of lipofectamine. Error bars represent errors between counts for three independent experiments. B) Immunofluorescence images showing the Cy3-positive cells are shown in red, and DAPI is shown in blue. Images were taken at 20x magnification. C) A barograph showing the percentages of Cy3-positive cells. Error bars represent errors between counts for three independent experiments. D) Immunofluorescence images showing cell densities following transfections, and the images were taken at 5x magnification. Cy3-positive cells are shown in red, and DAPI is shown in blue. [file 12935_2022_2814_MOESM5_ESM.tif]
