# Supplementary material for: The anterior gradient homologue 2 (AGR2) co-localises with the glucose-regulated protein 78 (GRP78) in cancer stem cells, and is critical for the survival and drug resistance of recurrent glioblastoma: in situ and in vitro analyses
Source: Cancer Cell Int. 2022 Dec 8;22:387. doi: 10.1186/s12935-022-02814-5 (PMC9730595; doi:10.1186/s12935-022-02814-5)
Supplement: Supplementary file 6 — Additional file 6: Table S1. Rare damaging COSMIC variants detected in the tissue and cell lines for Jed66_GB. [file 12935_2022_2814_MOESM6_ESM.docx]

**Supplementary Table 1.** Rare damaging COSMIC variants detected in the tissue and cell line for Jed66_GB.

| **Gene** | **Variant Reference** | **Consequence** | **Gene Information** |
| --- | --- | --- | --- |
| ABHD17A | NM_031213.3 , c.39delC , p.(Cys14AlafsTer43), NM_031213.3 , c.36_37delCT , p.(Phe13LeufsTer27) | Frameshift_Variants | Gene Name: Ubl carboxyl-terminal hydrolase 18; USP18; Family/Subfamily: UBL CARBOXYL-TERMINAL HYDROLASE 18-RELATED (PTHR24006:SF796), Protein Class: cysteine protease(PC00081), GO-Slim Molecular Function: thiol-dependent ubiquitin-specific protease activity(GO:0004843); cysteine-type endopeptidase activity(GO:0004197), GO-Slim Biological Process: protein deubiquitination(GO:0016579), Pathway: Interferon alpha/beta signaling; Post-translational protein modification; Interferon Signaling; Cytokine Signaling in Immune system; Metabolism of proteins; ISG15 antiviral mechanism; Antiviral mechanism by IFN-stimulated genes; Ub-specific processing proteases; Regulation of IFNA signaling; Immune System; Deubiquitination |
| ACAP1 | NM_014716.3 , c.1597C>T , p.(Arg533Trp) | Missense_Variant | Gene Name: Arf-GAP with coiled-coil, ANK repeat and PH domain-containing protein 1; ACAP1; Family/Subfamily: ARF-GAP WITH COILED-COIL, ANK REPEAT AND PH DOMAIN-CONTAINING PROTEIN 1 (PTHR23180:SF197) |
| ARID1B | NM_001346813.1 , c.942_944dupCGG , p.(Gly319dup) | Inframe_Insertion | Gene Name: AT-rich interactive domain-containing protein 1B; ARID1B; Family/Subfamily: AT-RICH INTERACTIVE DOMAIN-CONTAINING PROTEIN 1B (PTHR12656:SF11), GO-Slim Molecular Function: nucleosome binding(GO:0031491), GO-Slim Biological Process: positive regulation of transcription, DNA-templated(GO:0045893); ATP-dependent chromatin remodeling(GO:0043044); transcription by RNA polymerase II(GO:0006366); regulation of transcription by RNA polymerase II(GO:0006357), Pathway: Wnt signaling pathway->Switched/Sucrose Non Fermentation; ; , Reactome Pathway: Chromatin organization; Transcriptional regulation by RUNX1; RUNX1 interacts with co-factors whose precise effect on RUNX1 targets is not known; Chromatin modifying enzymes; RMTs methylate histone arginines; RNA Polymerase II Transcription; Generic Transcription Pathway; Gene expression (Transcription) |
| ARSD | NM_001669.3 , c.701_709delCCGGCGTGG , p.(Ala234_Val236del), NM_001669.3 , c.524G>A , p.(Gly175Asp) | Inframe_Deletion, Missense_Variant | Gene Name: Trypsin-3; PRSS3; Family/Subfamily: TRYPSIN-3 (PTHR24264:SF48), Protein Class: serine protease(PC00203), GO-Slim Molecular Function: serine-type endopeptidase activity(GO:0004252), GO-Slim Biological Process: proteolysis(GO:0006508), Pathway: Metabolism; Antimicrobial peptides; Metabolism of vitamins and cofactors; Defensins; Metabolism of water-soluble vitamins and cofactors; Neutrophil degranulation; Cobalamin (Cbl, vitamin B12) transport and metabolism; Immune System; Innate Immune System; Alpha-defensins |
| BCR | NM_004327.3 , c.3275_3278dupCCGG , p.(Val1094ArgfsTer17) | Frameshift_Variant | Gene Name: Mucosal addressin cell adhesion molecule 1; MADCAM1; Family/Subfamily: MUCOSAL ADDRESSIN CELL ADHESION MOLECULE 1 (PTHR14162:SF1), GO-Slim Molecular Function: integrin binding(GO:0005178), GO-Slim Biological Process: positive regulation of leukocyte migration(GO:0002687); integrin-mediated signaling pathway(GO:0007229); leukocyte migration(GO:0050900); leukocyte cell-cell adhesion(GO:0007159) |
| CD48 | NM_001256030.1 , c.58T>C , p.(Ser20Pro) | Missense_Variant | Gene Name: CD48 antigen; CD48; Family/Subfamily: CD48 ANTIGEN (PTHR12080:SF55), Protein Class: immunoglobulin receptor superfamily(PC00124), Reactome Pathway: Hemostasis; Cell surface interactions at the vascular wall |
| CDC42BPA | NM_003607.3 , c.3929G>A , p.(Arg1310His) | Missense_Variant | Gene Name: Alpha/beta hydrolase domain-containing protein 17A; ABHD17A; Family/Subfamily: ALPHA/BETA HYDROLASE DOMAIN-CONTAINING PROTEIN 17A (PTHR12277:SF52), Protein Class: serine protease(PC00203), GO-Slim Molecular Function: catalytic activity, acting on a protein(GO:0140096); thiolester hydrolase activity(GO:0016790), GO-Slim Biological Process: protein catabolic process(GO:0030163); lipoprotein metabolic process(GO:0042157); protein deacylation(GO:0035601) |
| CFAP100 | NM_182628.2 , c.589G>A , p.(Ala197Thr) | Missense_Variant | Gene Name: Nuclear factor erythroid 2-related factor 2; NFE2L2; Family/Subfamily: NUCLEAR FACTOR ERYTHROID 2-RELATED FACTOR 2 (PTHR24411:SF3), Protein Class: basic leucine zipper transcription factor(PC00056), GO-Slim Molecular Function: DNA-binding transcription factor activity(GO:0003700); transcription regulatory region sequence-specific DNA binding(GO:0000976), GO-Slim Biological Process: positive regulation of transcription, DNA-templated(GO:0045893); cellular response to oxidative stress(GO:0034599); transcription by RNA polymerase II(GO:0006366); regulation of transcription by RNA polymerase II(GO:0006357) |
| CRIPAK | NM_175918.3 , c.78_79insCACATGCCCATGTGGAGTGCCCGCCTGCTCA , p.(Cys27HisfsTer391) | Frameshift_Variant | Gene Name: Cysteine-rich PAK1 inhibitor; CRIPAK; ortholog |
| DENND3 | NM_014957.2 , c.2888C>T , p.(Ser963Leu) | Missense_Variant | Gene Name: DENN domain-containing protein 3; DENND3; Family/Subfamily: DENN DOMAIN-CONTAINING PROTEIN 3 (PTHR12296:SF28), GO-Slim Molecular Function: Rab guanyl-nucleotide exchange factor activity(GO:0017112), GO-Slim Biological Process: regulation of Ras protein signal transduction(GO:0046578); Ras protein signal transduction(GO:0007265), Reactome Pathway: Membrane Trafficking; Rab regulation of trafficking; Vesicle-mediated transport; RAB GEFs exchange GTP for GDP on RABs |
| DIS3L | NM_001143688.2 , c.2276G>A , p.(Arg759His) | Missense_Variant | Gene Name: DIS3-like exonuclease 1; DIS3L; Family/Subfamily: DIS3-LIKE EXONUCLEASE 1 (PTHR23355:SF30), Protein Class: exoribonuclease(PC00099), GO-Slim Molecular Function: 3'-5'-exoribonuclease activity(GO:0000175) |
| DNAJA4 | NM_018602.3 , c.379C>T , p.(Arg127Trp) | Missense_Variant | Gene Name: DnaJ homolog subfamily A member 4; DNAJA4; Family/Subfamily: DNAJ HOMOLOG SUBFAMILY A MEMBER 4 (PTHR43888:SF9), Protein Class: chaperone(PC00072), Reactome Pathway: Cellular responses to stress; Cellular responses to external stimuli; HSP90 chaperone cycle for steroid hormone receptors (SHR) |
| EPN2 | NM_014964.4 , c.1093C>A , p.(Pro365Thr) | Missense_Variant | Gene Name: Kinesin-like protein KIF26B; KIF26B; Family/Subfamily: KINESIN-LIKE PROTEIN KIF26B (PTHR24115:SF363), Protein Class: microtubule binding motor protein(PC00156), GO-Slim Molecular Function: ATPase activity(GO:0016887); microtubule motor activity(GO:0003777); microtubule binding(GO:0008017), GO-Slim Biological Process: microtubule-based movement(GO:0007018), Pathway: Membrane Trafficking; COPI-dependent Golgi-to-ER retrograde traffic; Vesicle-mediated transport; Golgi-to-ER retrograde transport; Hemostasis; Kinesins; Intra-Golgi and retrograde Golgi-to-ER traffic; Factors involved in megakaryocyte development and platelet production |
| FADS6 | NM_178128.5 , c.17_18insGATGGAACCTACGGAGCCCATGGAACCTACGGAGCCCATGGAACCTACGGAGCC , p.(Thr16_Pro33dup) | Inframe_Insertion | Gene Name: Fatty acid desaturase 6; FADS6; Family/Subfamily: FATTY ACID DESATURASE 6 (PTHR19353:SF13) |
| FAM214B | NM_025182.3 , c.11T>A , p.(Val4Glu) | Missense_Variant | Gene Name: Protein FAM214B; FAM214B; Family/Subfamily: PROTEIN FAM214B (PTHR13199:SF12) |
| KIF1A | NM_001244008.1 , c.3085G>A , p.(Val1029Met) | Missense_Variant | Gene Name: Epsin-2; EPN2; Family/Subfamily: EPSIN-2 (PTHR12276:SF50), GO-Slim Molecular Function: clathrin binding(GO:0030276); phospholipid binding(GO:0005543), GO-Slim Biological Process: vesicle budding from membrane(GO:0006900); membrane invagination(GO:0010324); endocytosis(GO:0006897), Pathway: Membrane Trafficking; Vesicle-mediated transport; Clathrin-mediated endocytosis; Cargo recognition for clathrin-mediated endocytosis |
| KIF26B | NM_018012.3 , c.3881C>T , p.(Ser1294Leu) | Missense_Variant | Gene Name: Breakpoint cluster region protein; BCR; Family/Subfamily: BREAKPOINT CLUSTER REGION PROTEIN (PTHR23182:SF3), Protein Class: G-protein modulator(PC00022), Pathway: Signal Transduction; Signaling by cytosolic FGFR1 fusion mutants; Signaling by Rho GTPases; Rho GTPase cycle; Signaling by FGFR1 in disease; Disease; Diseases of signal transduction; FGFR1 mutant receptor activation; Signaling by FGFR in disease |
| LNP1 | NM_001085451.1 , c.221_222insGGAATTCCGATGCCGATCGTCTGACCGTCTTCCTAGAAGGCATTCTCATGAGGACCA , p.(Ser80_His81insSerAspArgLeuProArgArgHisSerHisGluAspGlnGluPheArgCysArgSer) | Inframe_Insertion | Gene Name: Leukemia NUP98 fusion partner 1; LNP1; Family/Subfamily: LEUKEMIA NUP98 FUSION PARTNER 1 (PTHR35667:SF1) |
| LRIG3 | NM_153377.4 , c.1132A>G , p.(Met378Val) | Missense_Variant | Gene Name: Mucin-3A; MUC3A; Family/Subfamily: MUCIN-3A-RELATED (PTHR24041:SF22), Protein Class: cell adhesion molecule(PC00069), Pathway: Post-translational protein modification; C-type lectin receptors (CLRs); Dectin-2 family; Diseases of glycosylation; Termination of O-glycan biosynthesis; Defective GALNT12 causes colorectal cancer 1 (CRCS1); Metabolism of proteins; O-linked glycosylation of mucins; Diseases associated with O-glycosylation of proteins; Defective C1GALT1C1 causes Tn polyagglutination syndrome (TNPS); Disease; Immune System; O-linked glycosylation; Defective GALNT3 causes familial hyperphosphatemic tumoral calcinosis (HFTC); Innate Immune System |
| LTB4R | NM_001143919.2 , c.-108G>C , | 5_Prime_UTR_Variant | Gene Name: Leukotriene B4 receptor 1; LTB4R; Family/Subfamily: LEUKOTRIENE B4 RECEPTOR 1 (PTHR24230:SF51), Protein Class: G-protein coupled receptor(PC00021), GO-Slim Molecular Function: G protein-coupled peptide receptor activity(GO:0008528); peptide binding(GO:0042277), GO-Slim Biological Process: neuropeptide signaling pathway(GO:0007218), Pathway: , Reactome Pathway: G alpha (q) signalling events; Eicosanoid ligand-binding receptors; GPCR downstream signalling; Signaling by GPCR; Signal Transduction; GPCR ligand binding; Class A/1 (Rhodopsin-like receptors); Leukotriene receptors |
| MADCAM1 | NM_130760.2 , c.784_785insAGGAGCCTCCCGACACCACCTCCCAGGAGCCTCCCGACACCACCTCCC , p.(Ser261_Pro262insGlnGluProProAspThrThrSerGlnGluProProAspThrThrSer) | Inframe_Insertion | Gene Name: Arylsulfatase D; ARSD; Family/Subfamily: ARYLSULFATASE D (PTHR42693:SF12), Protein Class: hydrolase(PC00121), GO-Slim Molecular Function: hydrolase activity, acting on ester bonds(GO:0016788), Pathway: The activation of arylsulfatases; Glycosphingolipid metabolism; Metabolism of lipids; Gamma carboxylation, hypusine formation and arylsulfatase activation; Post-translational protein modification; Sphingolipid metabolism; Metabolism of proteins; Metabolism |
| MB21D2 | NM_178496.3 , c.688C>T , p.(Arg230Cys) | Missense_Variant | Gene Name: RING finger protein 225; RNF225; Family/Subfamily: RING FINGER PROTEIN 225 (PTHR22791:SF1) |
| MELTF | NM_005929.5 , c.1762G>A , p.(Glu588Lys) | Missense_Variant | Gene Name: Melanotransferrin; MELTF; Family/Subfamily: MELANOTRANSFERRIN (PTHR11485:SF21), Protein Class: transfer/carrier protein(PC00219), GO-Slim Biological Process: iron ion transport(GO:0006826), Reactome Pathway: Post-translational modification: synthesis of GPI-anchored proteins; Post-translational protein phosphorylation; Post-translational protein modification; Regulation of Insulin-like Growth Factor (IGF) transport and uptake by Insulin-like Growth Factor Binding Proteins (IGFBPs); Metabolism of proteins |
| MUC2 | NM_002457.4 , c.4422_4439delCACCACTCCCAGCCCTCC , p.(Pro1477_Thr1482del), NM_002457.4 , c.2891C>G , p.(Thr3439Ter) | Inframe_Deletion, Protein_Altering_Variant | Gene Name: Mucin-2; MUC2; Family/Subfamily: MUCIN-2 (PTHR11339:SF391), Protein Class: extracellular matrix protein(PC00102), Reactome Pathway: Post-translational protein modification; C-type lectin receptors (CLRs); Dectin-2 family; Diseases of glycosylation; Termination of O-glycan biosynthesis; Defective GALNT12 causes colorectal cancer 1 (CRCS1); Metabolism of proteins; O-linked glycosylation of mucins; Diseases associated with O-glycosylation of proteins; Defective C1GALT1C1 causes Tn polyagglutination syndrome (TNPS); Disease; Immune System; O-linked glycosylation; Defective GALNT3 causes familial hyperphosphatemic tumoral calcinosis (HFTC); Innate Immune System |
| MUC3A | NM_005960.1 , c.328A>C , p.(Ser1952Ter), NM_005960.1 , c.982G>A , p.(Thr2170Ter) | Protein_Altering_Variants | Gene Name: Leucine-rich repeats and immunoglobulin-like domains protein 3; LRIG3; Family/Subfamily: LEUCINE-RICH REPEATS AND IMMUNOGLOBULIN-LIKE DOMAINS PROTEIN 3 (PTHR24373:SF311), Protein Class: transmembrane signal receptor(PC00197) |
| NCOR1 | NM_006311.3 , c.4218A>C , p.(Leu1406Phe) | Missense_Variant | Gene Name: Nuclear receptor corepressor 1; NCOR1; Family/Subfamily: NUCLEAR RECEPTOR COREPRESSOR 1-RELATED (PTHR13992:SF5), Protein Class: chromatin/chromatin-binding, or -regulatory protein(PC00077), GO-Slim Molecular Function: nuclear hormone receptor binding(GO:0035257), Pathway: Huntington disease->Nuclear receptor co-repressor; ; , Reactome Pathway: Generic Transcription Pathway; Metabolism; Transcriptional regulation of white adipocyte differentiation; PPARA activates gene expression; Mitochondrial biogenesis; HCMV Infection; Circadian Clock; Gene expression (Transcription); Constitutive Signaling by NOTCH1 PEST Domain Mutants; Disease; RNA Polymerase II Transcription; Activation of HOX genes during differentiation; NR1D1 (REV-ERBA) represses gene expression; Signaling by NOTCH1 in Cancer; Signaling by NOTCH1 PEST Domain Mutants in Cancer; Activation of anterior HOX genes in hindbrain development during early embryogenesis; Developmental Biology; Signaling by NOTCH1; Downregulation of SMAD2/3:SMAD4 transcriptional activity; Organelle biogenesis and maintenance; Transcriptional activity of SMAD2/SMAD3:SMAD4 heterotrimer; Signal Transduction; Signaling by NOTCH; Notch-HLH transcription pathway; HDACs deacetylate histones; Pervasive developmental disorders; Chromatin modifying enzymes; HCMV Early Events; Loss of function of MECP2 in Rett syndrome; Signaling by TGF-beta Receptor Complex; NR1H2 and NR1H3-mediated signaling; Transcriptional activation of mitochondrial biogenesis; NR1H2 & NR1H3 regulate gene expression to control bile acid homeostasis; Metabolism of lipids; Nuclear Receptor transcription pathway; Signaling by ERBB4; Regulation of lipid metabolism by PPARalpha; NOTCH1 Intracellular Domain Regulates Transcription; Nuclear signaling by ERBB4; Signaling by TGF-beta family members; Constitutive Signaling by NOTCH1 HD+PEST Domain Mutants; Signaling by Receptor Tyrosine Kinases; Infectious disease; Signaling by Nuclear Receptors; Diseases of signal transduction; Chromatin organization; NR1H3 & NR1H2 regulate gene expression linked to cholesterol transport and efflux; Transcriptional Regulation by MECP2; Regulation of MECP2 expression and activity; Loss of MECP2 binding ability to the NCoR/SMRT complex; Signaling by NOTCH1 HD+PEST Domain Mutants in Cancer |
| NFE2L2 | NM_006164.4 , c.925C>T , p.(Leu309Phe) | Missense_Variant | Gene Name: Protein MB21D2; MB21D2; Family/Subfamily: PROTEIN MB21D2 (PTHR10656:SF47) |
| NPHP3 | NM_153240.4 , c.3629C>A , p.(Ser1210Tyr) | Missense_Variant | Gene Name: Serine/threonine-protein kinase MRCK alpha; CDC42BPA; Family/Subfamily: SERINE/THREONINE-PROTEIN KINASE MRCK ALPHA (PTHR22988:SF31), Protein Class: non-receptor serine/threonine protein kinase(PC00167), GO-Slim Molecular Function: protein serine/threonine kinase activity(GO:0004674), GO-Slim Biological Process: actomyosin structure organization(GO:0031032); peptidyl-threonine phosphorylation(GO:0018107) |
| OR51B2 | NM_033180.4 , c.46C>T , p.(Pro16Ser) | Missense_Variant | Gene Name: Olfactory receptor 51B2; OR51B2; Family/Subfamily: OLFACTORY RECEPTOR 51B2 (PTHR26450:SF164), Protein Class: transmembrane signal receptor(PC00197), Reactome Pathway: G alpha (s) signalling events; GPCR downstream signalling; Olfactory Signaling Pathway; Signaling by GPCR; Signal Transduction |
| OTOP1 | NM_177998.1 , c.1793G>C , p.(Arg598Pro) | Missense_Variant | Gene Name: Proton channel OTOP1; OTOP1; Family/Subfamily: PROTON CHANNEL OTOP1 (PTHR21522:SF19), Protein Class: transporter(PC00227) |
| PCNX2 | NM_014801.3 , c.1078G>A , p.(Asp360Asn) | Missense_Variant | Gene Name: Non-receptor tyrosine-protein kinase TYK2; TYK2; Family/Subfamily: NON-RECEPTOR TYROSINE-PROTEIN KINASE TYK2 (PTHR45807:SF6), Pathway: Interleukin-4 and Interleukin-13 signaling; Cytokine Signaling in Immune system; Interferon Signaling; RAF-independent MAPK1/3 activation; Signaling by Interleukins; Interleukin-27 signaling; IL-6-type cytokine receptor ligand interactions; Interferon alpha/beta signaling; MAPK3 (ERK1) activation; MAPK1/MAPK3 signaling; Regulation of IFNA signaling; Interleukin-12 signaling; Interleukin-20 family signaling; Interleukin-23 signaling; MAPK1 (ERK2) activation; Signal Transduction; Interleukin-35 Signalling; Interleukin-6 family signaling; Other interleukin signaling; Immune System; Interleukin-6 signaling; Interleukin-12 family signaling; MAPK family signaling cascades; Interleukin-10 signaling |
| PHLDB1 | NM_015157.3 , c.4076G>A , p.(Arg1359His) | Missense_Variant | Gene Name: Pleckstrin homology-like domain family B member 1; PHLDB1; Family/Subfamily: PLECKSTRIN HOMOLOGY-LIKE DOMAIN FAMILY B MEMBER 1 (PTHR12156:SF23), GO-Slim Biological Process: regulation of microtubule cytoskeleton organization(GO:0070507); microtubule cytoskeleton organization(GO:0000226) |
| PLA2G4D | NM_178034.3 , c.46-2delA , | Splice_Acceptor_Variant | Gene Name: Cytosolic phospholipase A2 delta; PLA2G4D; Family/Subfamily: CYTOSOLIC PHOSPHOLIPASE A2 DELTA (PTHR10728:SF31), Protein Class: phospholipase(PC00186), GO-Slim Molecular Function: phospholipase A2 activity(GO:0004623); calcium ion binding(GO:0005509); calcium-dependent phospholipid binding(GO:0005544), GO-Slim Biological Process: glycerophospholipid catabolic process(GO:0046475), Pathway: Angiogenesis->Cytosolic Phospholipase A2; ; , Reactome Pathway: Acyl chain remodelling of PC; Hydrolysis of LPC; Metabolism; Synthesis of PA; Acyl chain remodelling of PS; Glycerophospholipid biosynthesis; Metabolism of lipids; Acyl chain remodelling of PG; Phospholipid metabolism; Acyl chain remodelling of PI; Acyl chain remodelling of PE |
| POLD1 | NM_001308632.1 , c.2353G>A , p.(Val785Ile) | Missense_Variant | Gene Name: DNA polymerase delta catalytic subunit; POLD1; Family/Subfamily: DNA POLYMERASE DELTA CATALYTIC SUBUNIT (PTHR10322:SF23), Protein Class: DNA metabolism protein(PC00009), Pathway: DNA replication->Pol delta; ; , Reactome Pathway: Cytosolic iron-sulfur cluster assembly; S Phase; Gap-filling DNA repair synthesis and ligation in TC-NER; Metabolism; Mismatch repair (MMR) directed by MSH2:MSH3 (MutSbeta); Global Genome Nucleotide Excision Repair (GG-NER); Nucleotide Excision Repair; Gap-filling DNA repair synthesis and ligation in GG-NER; Mismatch Repair; DNA Replication; Mismatch repair (MMR) directed by MSH2:MSH6 (MutSalpha); Chromosome Maintenance; Base Excision Repair; Synthesis of DNA; Telomere C-strand (Lagging Strand) Synthesis; Processive synthesis on the C-strand of the telomere; Polymerase switching on the C-strand of the telomere; Termination of translesion DNA synthesis; Leading Strand Synthesis; Dual incision in TC-NER; Recognition of DNA damage by PCNA-containing replication complex; Removal of the Flap Intermediate; Translesion synthesis by Y family DNA polymerases bypasses lesions on DNA template; Resolution of AP sites via the multiple-nucleotide patch replacement pathway; Transcription-Coupled Nucleotide Excision Repair (TC-NER); DNA strand elongation; PCNA-Dependent Long Patch Base Excision Repair; Homology Directed Repair; HDR through Homologous Recombination (HRR) or Single Strand Annealing (SSA); DNA Double-Strand Break Repair; Polymerase switching; Removal of the Flap Intermediate from the C-strand; Extension of Telomeres; Resolution of Abasic Sites (AP sites); Lagging Strand Synthesis; Processive synthesis on the lagging strand; Cell Cycle; DNA Repair; HDR through Homologous Recombination (HRR); DNA Damage Bypass; Cell Cycle, Mitotic; Dual Incision in GG-NER; Telomere Maintenance |
| POTEH | NM_001136213.1 , c.348C>G , p.(Asn116Lys) | Missense_Variant | Gene Name: POTE ankyrin domain family member H; POTEH; Family/Subfamily: POTE ANKYRIN DOMAIN FAMILY MEMBER E-RELATED (PTHR24118:SF94), Protein Class: membrane traffic protein(PC00150) |
| PRSS3 | NM_007343.3 , c.244G>A , p.(Val82Ile) | Missense_Variant | Gene Name: Tyrosine-protein phosphatase non-receptor type 5; PTPN5; Family/Subfamily: TYROSINE-PROTEIN PHOSPHATASE NON-RECEPTOR TYPE 5 (PTHR46198:SF1), Pathway: Immune System; Interleukin-1 family signaling; Interleukin-37 signaling; Signaling by Interleukins; Cytokine Signaling in Immune system |
| PTPN5 | NM_006906.1 , c.1519A>C , p.(Ile507Leu) | Missense_Variant | Gene Name: Pecanex-like protein 2; PCNX2; Family/Subfamily: PECANEX-LIKE PROTEIN 2 (PTHR12372:SF5) |
| RNF225 | NM_001195135.1 , c.132_134delGGA , p.(Glu45del) | Inframe_Deletion | Gene Name: Cilia- and flagella-associated protein 100; CFAP100; Family/Subfamily: CILIA- AND FLAGELLA-ASSOCIATED PROTEIN 100 (PTHR21683:SF5) |
| RP1L1 | NM_178857.5 , c.3922_3927delGAAGGA , p.(Glu1308_Gly1309del) | Inframe_Deletion | Gene Name: Retinitis pigmentosa 1-like 1 protein; RP1L1; Family/Subfamily: RETINITIS PIGMENTOSA 1-LIKE 1 PROTEIN (PTHR23005:SF3), GO-Slim Biological Process: retina development in camera-type eye(GO:0060041); intraciliary transport involved in cilium assembly(GO:0035735); vesicle targeting, trans-Golgi to periciliary membrane compartment(GO:0097712); axoneme assembly(GO:0035082); ciliary transition zone assembly(GO:1905349); neuron development(GO:0048666); protein localization to cilium(GO:0061512) |
| SELP | NM_003005.3 , c.283C>T , p.(Arg95Ter) | Stop_Gained | Gene Name: P-selectin; SELP; Family/Subfamily: P-SELECTIN (PTHR19325:SF484), Reactome Pathway: Response to elevated platelet cytosolic Ca2+; Hemostasis; Platelet activation, signaling and aggregation; Cell surface interactions at the vascular wall; Platelet degranulation |
| SEPT6 | NM_015129.5 , c.1202C>T , p.(Thr401Met) | Missense_Variant | Gene Name: Septin-6; SEPTIN6; Family/Subfamily: SEPTIN-6 (PTHR18884:SF55), Protein Class: cytoskeletal protein(PC00085), GO-Slim Molecular Function: GTPase activity(GO:0003924); binding(GO:0005488); molecular adaptor activity(GO:0060090), GO-Slim Biological Process: cytoskeleton-dependent cytokinesis(GO:0061640); membrane fission(GO:0090148); intraciliary transport involved in cilium assembly(GO:0035735); vesicle targeting, trans-Golgi to periciliary membrane compartment(GO:0097712); axoneme assembly(GO:0035082); ciliary transition zone assembly(GO:1905349); protein localization to cilium(GO:0061512) |
| SORD | NM_003104.5 , c.286C>T , p.(Pro96Ser) | Missense_Variant | Gene Name: Sorbitol dehydrogenase; SORD; Family/Subfamily: SORBITOL DEHYDROGENASE (PTHR43161:SF9), Protein Class: dehydrogenase(PC00092), Reactome Pathway: Fructose biosynthesis; Fructose metabolism; Metabolism of carbohydrates; Formation of xylulose-5-phosphate; Metabolism |
| SSTR3 | NM_001051.4 , c.1006C>T , p.(Arg336Cys) | Missense_Variant | Gene Name: Somatostatin receptor type 3; SSTR3; Family/Subfamily: SOMATOSTATIN RECEPTOR TYPE 3 (PTHR24229:SF42), Protein Class: G-protein coupled receptor(PC00021), GO-Slim Molecular Function: G protein-coupled receptor activity(GO:0004930); neuropeptide binding(GO:0042923), GO-Slim Biological Process: neuropeptide signaling pathway(GO:0007218), Pathway: Heterotrimeric G-protein signaling pathway-Gi alpha and Gs alpha mediated pathway->Gs-protein coupled receptor; ; Heterotrimeric G-protein signaling pathway-Gi alpha and Gs alpha mediated pathway->Gi protein coupled receptor; ; Heterotrimeric G-protein signaling pathway-Gq alpha and Go alpha mediated pathway->Go-protein coupled receptor, Reactome Pathway: Signal Transduction; Organelle biogenesis and maintenance; Peptide ligand-binding receptors; Cilium Assembly; Class A/1 (Rhodopsin-like receptors); G alpha (i) signalling events; GPCR ligand binding; BBSome-mediated cargo-targeting to cilium; Signaling by GPCR; Cargo trafficking to the periciliary membrane; GPCR downstream signalling |
| STC2 | NM_003714.2 , c.877G>A , p.(Glu293Lys) | Missense_Variant | Gene Name: Stanniocalcin-2; STC2; Family/Subfamily: STANNIOCALCIN-2 (PTHR11245:SF2), Protein Class: peptide hormone(PC00179), GO-Slim Biological Process: cellular calcium ion homeostasis(GO:0006874), Reactome Pathway: Post-translational protein phosphorylation; Post-translational protein modification; Regulation of Insulin-like Growth Factor (IGF) transport and uptake by Insulin-like Growth Factor Binding Proteins (IGFBPs); Metabolism of proteins |
| SUSD2 | NM_019601.3 , c.209_210invGA , p.(Gly70Val) | Missense_Variant | Gene Name: Sushi domain-containing protein 2; SUSD2; Family/Subfamily: SUSHI DOMAIN-CONTAINING PROTEIN 2 (PTHR13802:SF52) |
| TBC1D26 | NM_178571.4 , c.410G>T , p.(Arg137Met) | Missense_Variant | Gene Name: TBC1 domain family member 26; TBC1D26; Family/Subfamily: TBC1 DOMAIN FAMILY MEMBER 26-RELATED (PTHR22957:SF598), Protein Class: GTPase-activating protein(PC00257), GO-Slim Molecular Function: GTPase activity(GO:0003924); GTPase activator activity(GO:0005096); Rab GTPase binding(GO:0017137), GO-Slim Biological Process: intracellular protein transport(GO:0006886); positive regulation of GTPase activity(GO:0043547) |
| TPI1 | NM_001159287.1 , c.728G>A , p.(Arg243His) | Missense_Variant | Gene Name: Triosephosphate isomerase; TPI1; Family/Subfamily: TRIOSEPHOSPHATE ISOMERASE (PTHR21139:SF24), Protein Class: isomerase(PC00135), GO-Slim Molecular Function: isomerase activity(GO:0016853), GO-Slim Biological Process: small molecule biosynthetic process(GO:0044283); small molecule catabolic process(GO:0044282); carbohydrate biosynthetic process(GO:0016051); cellular carbohydrate catabolic process(GO:0044275); glycolytic process(GO:0006096); alcohol metabolic process(GO:0006066); oxidation-reduction process(GO:0055114); carbohydrate derivative biosynthetic process(GO:1901137); cellular biosynthetic process(GO:0044249); glucose metabolic process(GO:0006006); organophosphate biosynthetic process(GO:0090407), Pathway: Glycolysis->Triosephosphate isomerase, Reactome Pathway: Glycolysis; Gluconeogenesis; Metabolism of carbohydrates; Glucose metabolism; Metabolism |
| TRAP1 | NM_016292.2 , c.1406G>A , p.(Arg469His) | Missense_Variant | Gene Name: Putative endoplasmin-like protein; HSP90B2P; |
| TRARG1 | NM_172367.2 , c.317T>C , p.(Ile106Thr) | Missense_Variant | Gene Name: Trafficking regulator of GLUT4 (SLC2A4) 1 |
| TYK2 | NM_003331.4 , c.1807G>A , p.(Val603Met) | Missense_Variant | Gene Name: Nephrocystin-3; NPHP3; Family/Subfamily: NEPHROCYSTIN-3 (PTHR45641:SF10), Pathway: Cilium Assembly; Cargo trafficking to the periciliary membrane; Trafficking of myristoylated proteins to the cilium; Organelle biogenesis and maintenance |
| USP18 | NM_017414.3 , c.907G>A , p.(Glu303Lys) | Missense_Variant | Gene Name: Kinesin-like protein KIF1A; KIF1A; Family/Subfamily: KINESIN-LIKE PROTEIN KIF1A (PTHR24115:SF361), Protein Class: microtubule binding motor protein(PC00156), GO-Slim Molecular Function: ATP-dependent microtubule motor activity, plus-end-directed(GO:0008574); microtubule binding(GO:0008017), GO-Slim Biological Process: vesicle-mediated transport(GO:0016192); cytoskeleton-dependent intracellular transport(GO:0030705); microtubule-based movement(GO:0007018), Pathway: Membrane Trafficking; COPI-dependent Golgi-to-ER retrograde traffic; Vesicle-mediated transport; Golgi-to-ER retrograde transport; Hemostasis; Kinesins; Intra-Golgi and retrograde Golgi-to-ER traffic; Factors involved in megakaryocyte development and platelet production |

Only variants with a possible/probable damaging PolyPhen effect were included, as per data annotated by BaseSpace or detected manually using PolyPhen-2 Wiki.
