# Supplementary material for: The anterior gradient homologue 2 (AGR2) co-localises with the glucose-regulated protein 78 (GRP78) in cancer stem cells, and is critical for the survival and drug resistance of recurrent glioblastoma: in situ and in vitro analyses
Source: Cancer Cell Int. 2022 Dec 8;22:387. doi: 10.1186/s12935-022-02814-5 (PMC9730595; doi:10.1186/s12935-022-02814-5)
Supplement: Supplementary file 7 — Additional file 7: Table S2. Rare damaging COSMIC variants detected in the tissue and cell lines for Jed41_GB. [file 12935_2022_2814_MOESM7_ESM.docx]

**Supplementary Table 2.** Rare damaging COSMIC variants detected in the tissue and cell line for Jed41_GB.

| **Gene** | **Variant Reference** | **Consequence** | **Gene Information** |
| --- | --- | --- | --- |
| ADGRE5 | NM_078481.3 , c.1892G>A , p.(Ser631Asn) | missense_variant | Gene Name: Adhesion G protein-coupled receptor E5; ADGRE5, Family/Subfamily: ADHESION G PROTEIN-COUPLED RECEPTOR E5 (PTHR12011:SF348), Protein Class: G-protein coupled receptor(PC00021), GO-Slim Molecular Function: G protein-coupled receptor activity(GO:0004930), GO-Slim Biological Process: regulation of cAMP-mediated signaling(GO:0043949); regulation of adenylate cyclase activity(GO:0045761); adenylate cyclase-activating G protein-coupled receptor signaling pathway(GO:0007189); activation of adenylate cyclase activity(GO:0007190), Pathway: Immune System; Signaling by GPCR; Signal Transduction; Class B/2 (Secretin family receptors); Innate Immune System; GPCR ligand binding; Neutrophil degranulation |
| ADGRF4 | NM_153838.4 , c.1009G>A , p.(Glu337Lys) | missense_variant | Gene Name: Adhesion G protein-coupled receptor F4; ADGRF4, Family/Subfamily: ADHESION G PROTEIN-COUPLED RECEPTOR F4 (PTHR45813:SF1) |
| ATG2B | NM_018036.6 , c.4639C>T , p.(Arg1547Cys) | missense_variant | Gene Name: Autophagy-related protein 2 homolog B; ATG2B, Family/Subfamily: AUTOPHAGY-RELATED PROTEIN 2 HOMOLOG B (PTHR13190:SF20), GO-Slim Molecular Function: phosphatidylinositol-3-phosphate binding(GO:0032266), GO-Slim Biological Process: autophagosome assembly(GO:0000045); piecemeal microautophagy of the nucleus(GO:0034727); proteolysis(GO:0006508); endoplasmic reticulum organization(GO:0007029); autophagy of mitochondrion(GO:0000422); nucleus organization(GO:0006997) |
| ATN1 | NM_001007026.1 , c.1491_1508delGCAGCAGCAGCAGCAGCA , p.(Gln497_Gln502del) | inframe_deletion | Gene Name: Atrophin-1; ATN1, Family/Subfamily: ATROPHIN-1 (PTHR13859:SF9), GO-Slim Molecular Function: RNA polymerase II transcription factor binding(GO:0001085); transcription corepressor activity(GO:0003714), Pathway: Regulation of PTEN gene transcription; PTEN Regulation; Signal Transduction; Intracellular signaling by second messengers; PIP3 activates AKT signaling |
| CASP8AP2 | NM_012115.3 , c.440G>A , p.(Arg147Gln) | missense_variant | Gene Name: CASP8-associated protein 2; CASP8AP2, Family/Subfamily: CASP8-ASSOCIATED PROTEIN 2 (PTHR15489:SF2), Protein Class: protein-binding activity modulator(PC00095), GO-Slim Molecular Function: transcription corepressor activity(GO:0003714), GO-Slim Biological Process: proteolysis(GO:0006508); cellular protein metabolic process(GO:0044267); activation of cysteine-type endopeptidase activity involved in apoptotic process(GO:0006919); extrinsic apoptotic signaling pathway via death domain receptors(GO:0008625), Reactome Pathway: SUMOylation; SUMO E3 ligases SUMOylate target proteins; Post-translational protein modification; SUMOylation of transcription cofactors; Metabolism of proteins |
| CCDC40 | NM_017950.3 , c.850G>C , p.(Asp284His) | missense_variant | Gene Name: Coiled-coil domain-containing protein 40; CCDC40, Family/Subfamily: COILED-COIL DOMAIN-CONTAINING PROTEIN 40 (PTHR16275:SF8) |
| CCNF | NM_001761.2 , c.220G>A , p.(Ala74Thr) | missense_variant | Gene Name: Cyclin-F; CCNF, Family/Subfamily: CYCLIN-F (PTHR10177:SF496), Protein Class: kinase activator(PC00138), GO-Slim Molecular Function: protein kinase binding(GO:0019901); cyclin-dependent protein serine/threonine kinase regulator activity(GO:0016538); cyclin binding(GO:0030332); cyclin-dependent protein serine/threonine kinase activity(GO:0004693), GO-Slim Biological Process: mitotic nuclear division(GO:0140014); mitotic cell cycle phase transition(GO:0044772); protein phosphorylation(GO:0006468); regulation of cyclin-dependent protein serine/threonine kinase activity(GO:0000079), Pathway: Class I MHC mediated antigen processing & presentation; Immune System; Antigen processing: Ubiquitination & Proteasome degradation; Adaptive Immune System; Neddylation; Post-translational protein modification; Metabolism of proteins |
| CERCAM | NM_016174.4 , c.1172G>C , p.(Cys391Ser) | missense_variant | Gene Name: Inactive glycosyltransferase 25 family member 3; CERCAM, Family/Subfamily: INACTIVE GLYCOSYLTRANSFERASE 25 FAMILY MEMBER 3 (PTHR10730:SF9), Protein Class: protein modifying enzyme(PC00260), GO-Slim Molecular Function: UDP-glycosyltransferase activity(GO:0008194); catalytic activity, acting on a protein(GO:0140096); galactosyltransferase activity(GO:0008378) |
| DMXL1 | NM_001290321.2 , c.6167G>A , p.(Arg2056His) | missense_variant | Gene Name: DmX-like protein 1; DMXL1, Family/Subfamily: DMX-LIKE PROTEIN 1 (PTHR13950:SF12), GO-Slim Biological Process: vacuolar acidification(GO:0007035) |
| FADS6 | NM_178128.5 , c.17_18insGATGGAACCTACGGAGCCCATGGAACCTACGGAGCCCATGGAACCTACGGAGCC , p.(Thr16_Pro33dup) | inframe_insertion | Gene Name: Fatty acid desaturase 6; FADS6, Family/Subfamily: FATTY ACID DESATURASE 6 (PTHR19353:SF13), |
| FAM187B | NM_152481.1 , c.301C>T , p.(Arg101Cys) | missense_variant | Gene Name: Protein FAM187B; FAM187B, Family/Subfamily: PROTEIN FAM187B (PTHR32178:SF8), Protein Class: immunoglobulin superfamily cell adhesion molecule(PC00125) |
| FN3K | NM_022158.3 , c.232G>A , p.(Asp78Asn) | missense_variant | Gene Name: Fructosamine-3-kinase; FN3K, Family/Subfamily: FRUCTOSAMINE-3-KINASE (PTHR12149:SF9), Protein Class: kinase(PC00137), GO-Slim Molecular Function: kinase activity(GO:0016301), GO-Slim Biological Process: amino sugar metabolic process(GO:0006040), Reactome Pathway: Gamma carboxylation, hypusine formation and arylsulfatase activation; Post-translational protein modification; Metabolism of proteins |
| HDDC3 | NM_001286451.1 , c.531_533dupGAC , p.(Thr178dup) | inframe_insertion | Gene Name: Guanosine-3',5'-bis(diphosphate) 3'-pyrophosphohydrolase MESH1; HDDC3, Family/Subfamily: GUANOSINE-3',5'-BIS(DIPHOSPHATE) 3'-PYROPHOSPHOHYDROLASE MESH1 (PTHR46246:SF1), Protein Class: hydrolase(PC00121), GO-Slim Molecular Function: phosphoric ester hydrolase activity(GO:0042578) |
| KCNK17 | NM_031460.3 , c.324_326delCTT , p.(Phe109del) | inframe_deletion | Gene Name: Potassium channel subfamily K member 17; KCNK17, Family/Subfamily: POTASSIUM CHANNEL SUBFAMILY K MEMBER 17 (PTHR11003:SF319), Protein Class: ion channel(PC00133), GO-Slim Molecular Function: potassium channel activity(GO:0005267), GO-Slim Biological Process: potassium ion transmembrane transport(GO:0071805); regulation of membrane potential(GO:0042391), Pathway: Cardiac conduction; Phase 4 - resting membrane potential; TWIK-related alkaline pH activated K+ channel (TALK); Tandem pore domain potassium channels; Potassium Channels; Neuronal System; Muscle contraction |
| LIPF | NM_001198829.1 , c.851G>A , p.(Arg284His) | missense_variant | Gene Name: Gastric triacylglycerol lipase; LIPF, Family/Subfamily: GASTRIC TRIACYLGLYCEROL LIPASE (PTHR11005:SF15), Protein Class: lipase(PC00143), Pathway: Digestion; Digestion of dietary lipid; Digestion and absorption |
| LNP1 | NM_001085451.1 , c.221_222insGGAATTCCGATGCCGATCGTCTGACCGTCTTCCTAGAAGGCATTCTCATGAGGACCA , p.(Ser80_His81insSerAspArgLeuProArgArgHisSerHisGluAspGlnGluPheArgCysArgSer) | inframe_insertion | Gene Name: Leukemia NUP98 fusion partner 1; LNP1, Family/Subfamily: LEUKEMIA NUP98 FUSION PARTNER 1 (PTHR35667:SF1) |
| LRP1B | NM_018557.2 , c.12161A>C , p.(Glu4054Ala) | missense_variant | Gene Name: Low-density lipoprotein receptor-related protein 1B; LRP1B, Family/Subfamily: LOW-DENSITY LIPOPROTEIN RECEPTOR-RELATED PROTEIN 1B (PTHR22722:SF5), Pathway: Alzheimer disease-presenilin pathway->LRP N-terminal fragment; Alzheimer disease-presenilin pathway->Low-density lipoprotein receptor-related protein; Alzheimer disease-presenilin pathway->LRP intracellular fragment; Alzheimer disease-presenilin pathway->LRP C-terminal fragment; Alzheimer disease-presenilin pathway->LRP transmembrane fragment |
| MAZ | NM_001042539.2 , c.*57_*59dupGGC , | 3_prime_UTR_variant | Gene Name: Myc-associated zinc finger protein; MAZ, Family/Subfamily: MYC-ASSOCIATED ZINC FINGER PROTEIN (PTHR24390:SF122), Protein Class: C2H2 zinc finger transcription factor(PC00248), GO-Slim Molecular Function: RNA polymerase II cis-regulatory region sequence-specific DNA binding(GO:0000978); DNA-binding transcription factor activity, RNA polymerase II-specific(GO:0000981), GO-Slim Biological Process: transcription by RNA polymerase II(GO:0006366); regulation of transcription by RNA polymerase II(GO:0006357) |
| MISP3 | NM_001291291.1 , c.48G>T , p.(Glu16Asp) | missense_variant | Gene Name: Uncharacterized protein MISP3; MISP3, Family/Subfamily: MISP FAMILY MEMBER 3 (PTHR18839:SF4) |
| MUC2 | NM_002457.4 , c.4422_4439delCACCACTCCCAGCCCTCC , p.(Pro1477_Thr1482del),NM_002457.4 , c.2947C>A , p.(Thr3458Ter) | inframe_deletion, protein_altering_variant | Gene Name: Mucin-2; MUC2, Family/Subfamily: MUCIN-2 (PTHR11339:SF391), Protein Class: extracellular matrix protein(PC00102), Reactome Pathway: Post-translational protein modification; C-type lectin receptors (CLRs); Dectin-2 family; Diseases of glycosylation; Termination of O-glycan biosynthesis; Defective GALNT12 causes colorectal cancer 1 (CRCS1); Metabolism of proteins; O-linked glycosylation of mucins; Diseases associated with O-glycosylation of proteins; Defective C1GALT1C1 causes Tn polyagglutination syndrome (TNPS); Disease; Immune System; O-linked glycosylation; Defective GALNT3 causes familial hyperphosphatemic tumoral calcinosis (HFTC); Innate Immune System |
| MUC3A | NM_005960.1 , c.328A>C , p.(Ser1952Ter),NM_005960.1 , c.982G>A , p.(Thr2170Ter) | protein_altering_variant | Gene Name: Mucin-3A; MUC3A, Family/Subfamily: MUCIN-3A-RELATED (PTHR24041:SF22), Protein Class: cell adhesion molecule(PC00069), Pathway: Post-translational protein modification; C-type lectin receptors (CLRs); Dectin-2 family; Diseases of glycosylation; Termination of O-glycan biosynthesis; Defective GALNT12 causes colorectal cancer 1 (CRCS1); Metabolism of proteins; O-linked glycosylation of mucins; Diseases associated with O-glycosylation of proteins; Defective C1GALT1C1 causes Tn polyagglutination syndrome (TNPS); Disease; Immune System; O-linked glycosylation; Defective GALNT3 causes familial hyperphosphatemic tumoral calcinosis (HFTC); Innate Immune System |
| NFE2L2 | NM_006164.4 , c.925C>T , p.(Leu309Phe) | missense_variant | Gene Name: Nuclear factor erythroid 2-related factor 2; NFE2L2, Family/Subfamily: NUCLEAR FACTOR ERYTHROID 2-RELATED FACTOR 2 (PTHR24411:SF3), Protein Class: basic leucine zipper transcription factor(PC00056), GO-Slim Molecular Function: DNA-binding transcription factor activity(GO:0003700); transcription regulatory region sequence-specific DNA binding(GO:0000976), GO-Slim Biological Process: positive regulation of transcription, DNA-templated(GO:0045893); cellular response to oxidative stress(GO:0034599); transcription by RNA polymerase II(GO:0006366); regulation of transcription by RNA polymerase II(GO:0006357) |
| OR2T11 | NM_001001964.1 , c.608T>G , p.(Met203Arg) | missense_variant | Gene Name: Olfactory receptor 2T11; OR2T11, Family/Subfamily: OLFACTORY RECEPTOR 2T11 (PTHR26453:SF173), Protein Class: transmembrane signal receptor(PC00197), Reactome Pathway: G alpha (s) signalling events; GPCR downstream signalling; Olfactory Signaling Pathway; Signaling by GPCR; Signal Transduction |
| OR2T33 | NM_001004695.1 , c.692C>G , p.(Ala231Gly) | missense_variant | Gene Name: Olfactory receptor 2T33; OR2T33, Family/Subfamily: OLFACTORY RECEPTOR 2T33-RELATED (PTHR26453:SF286), Protein Class: transmembrane signal receptor(PC00197), Reactome Pathway: G alpha (s) signalling events; GPCR downstream signalling; Olfactory Signaling Pathway; Signaling by GPCR; Signal Transduction |
| OR3A1 | NM_002550.2 , c.762C>A , p.(Phe254Leu) | missense_variant | Gene Name: Olfactory receptor 3A1; OR3A1, Family/Subfamily: OLFACTORY RECEPTOR 3A1 (PTHR48001:SF7), Protein Class: transmembrane signal receptor(PC00197), Reactome Pathway: G alpha (s) signalling events; GPCR downstream signalling; Olfactory Signaling Pathway; Signaling by GPCR; Signal Transduction |
| PHLDB3 | NM_198850.3 , c.559C>T , p.(Arg187Trp) | missense_variant | Gene Name: Pleckstrin homology-like domain family B member 3; PHLDB3, Family/Subfamily: PLECKSTRIN HOMOLOGY-LIKE DOMAIN FAMILY B MEMBER 3 (PTHR12156:SF22) |
| PRSS45 | NM_199183.2 , c.40G>T , p.(Glu14Ter) | stop_gained | Gene Name: Putative serine protease 45; PRSS45P, Family/Subfamily: SERINE PROTEASE 45-RELATED (PTHR24256:SF140), Protein Class: serine protease, GO-Slim Molecular Function: serine-type endopeptidase activity, GO-Slim Biological Process: proteolysis |
| RCOR3 | NM_001136223.1 , c.607C>T , p.(Arg203Cys) | missense_variant | Gene Name: REST corepressor 3; RCOR3, Family/Subfamily: REST COREPRESSOR 3 (PTHR16089:SF13), GO-Slim Molecular Function: transcription corepressor activity(GO:0003714), GO-Slim Biological Process: negative regulation of transcription, DNA-templated(GO:0045892); transcription by RNA polymerase II(GO:0006366); histone deacetylation(GO:0016575); regulation of transcription by RNA polymerase II(GO:0006357) |
| STAB2 | NM_017564.9 , c.6061G>A , p.(Asp2021Asn) | missense_variant | Gene Name: Stabilin-2; STAB2, Family/Subfamily: STABILIN-2 (PTHR24038:SF0), Protein Class: membrane trafficking regulatory protein(PC00151), Reactome Pathway: Vesicle-mediated transport; Glycosaminoglycan metabolism; Hyaluronan uptake and degradation; Binding and Uptake of Ligands by Scavenger Receptors; Metabolism of carbohydrates; Scavenging by Class H Receptors; Hyaluronan metabolism; Metabolism |
| SUN2 | NM_001199579.1 , c.743C>T , p.(Thr248Met) | missense_variant | Gene Name: Sad1 and UNC84 domain containing 2; SUN2, Family/Subfamily: SUN DOMAIN-CONTAINING PROTEIN 2 (PTHR12911:SF22), Protein Class: non-motor microtubule binding protein(PC00166), GO-Slim Molecular Function: protein binding(GO:0005515); protein-macromolecule adaptor activity(GO:0030674), GO-Slim Biological Process: nuclear envelope organization(GO:0006998), Reactome Pathway: Cell Cycle; Reproduction; Meiosis; Meiotic synapsis |
| SUSD2 | NM_019601.3 , c.209_210invGA , p.(Gly70Val) | missense_variant | Gene Name: Sushi domain-containing protein 2; SUSD2, Family/Subfamily: SUSHI DOMAIN-CONTAINING PROTEIN 2 (PTHR13802:SF52) |
| TP53 | NM_000546.5 , c.817C>T , p.(Arg273Cys) | missense_variant | Gene Name: Cellular tumor antigen p53; TP53, Family/Subfamily: CELLULAR TUMOR ANTIGEN P53 (PTHR11447:SF6), Protein Class: P53-like transcription factor(PC00253), GO-Slim Molecular Function: RNA polymerase II cis-regulatory region sequence-specific DNA binding(GO:0000978); DNA-binding transcription factor activity, RNA polymerase II-specific(GO:0000981), GO-Slim Biological Process: transcription by RNA polymerase II(GO:0006366); regulation of transcription by RNA polymerase II(GO:0006357), Pathway: p53 pathway by glucose deprivation->Tumor protein p53; p53 pathway->Tumor protein p53; p53 pathway->Tumor protein p53; P53 pathway feedback loops 1->Tumor protein p53; Huntington disease->Tumor protein p53; Apoptosis signaling pathway->Tumor protein p53; ; p53 pathway feedback loops 2->Tumor protein p53; Wnt signaling pathway->Tumor protein p53; Reactome Pathway: Activation of BH3-only proteins; Intracellular signaling by second messengers; Signaling by NOTCH; Intrinsic Pathway for Apoptosis; Activation of NOXA and translocation to mitochondria; Stabilization of p53; Ovarian tumor domain proteases; Metabolism of proteins; SUMOylation of transcription factors; PI5P Regulates TP53 Acetylation; TP53 Regulates Transcription of Death Receptors and Ligands; Transcriptional activation of cell cycle inhibitor p21; TP53 Regulates Transcription of Genes Involved in G1 Cell Cycle Arrest; TP53 regulates transcription of additional cell cycle genes whose exact role in the p53 pathway remain uncertain; TP53 Regulates Transcription of Genes Involved in G2 Cell Cycle Arrest; TP53 Regulates Metabolic Genes; TP53 Regulates Transcription of Cell Cycle Genes; Regulation of TP53 Activity through Methylation; Immune System; RUNX3 regulates CDKN1A transcription; Activation of PUMA and translocation to mitochondria; PIP3 activates AKT signaling; DNA Double Strand Break Response; TP53 Regulates Transcription of Caspase Activators and Caspases; TP53 regulates transcription of several additional cell death genes whose specific roles in p53-dependent apoptosis remain uncertain; TP53 Regulates Transcription of Genes Involved in Cytochrome C Release; Ub-specific processing proteases; Signaling by Interleukins; Regulation of TP53 Activity through Association with Co-factors; Regulation of TP53 Activity through Acetylation; Regulation of TP53 Degradation; Deubiquitination; Recruitment and ATM-mediated phosphorylation of repair and signaling proteins at DNA double strand breaks; Regulation of TP53 Activity through Phosphorylation; Factors involved in megakaryocyte development and platelet production; PTEN Regulation; Regulation of TP53 Expression; SUMO E3 ligases SUMOylate target proteins; DNA Damage/Telomere Stress Induced Senescence; Oncogene Induced Senescence; Autodegradation of the E3 ubiquitin ligase COP1; Cell Cycle Checkpoints; Formation of Senescence-Associated Heterochromatin Foci (SAHF); Cellular Senescence; Oxidative Stress Induced Senescence; Association of TriC/CCT with target proteins during biosynthesis; Regulation of TP53 Expression and Degradation; Generic Transcription Pathway; p53-Dependent G1/S DNA damage checkpoint; Mitotic G2-G2/M phases; Cellular responses to external stimuli; RNA Polymerase II Transcription; Interleukin-4 and Interleukin-13 signaling; Cytokine Signaling in Immune system; G2/M Checkpoints; Transcriptional Regulation by VENTX; Signal Transduction; G1/S DNA Damage Checkpoints; Chaperonin-mediated protein folding; Post-translational protein modification; SUMOylation; Gene expression (Transcription); TP53 Regulates Transcription of Cell Death Genes; Regulation of TP53 Activity; Programmed Cell Death; G2/M DNA damage checkpoint; Cell Cycle, Mitotic; G2/M Transition; TP53 Regulates Transcription of DNA Repair Genes; Pre-NOTCH Expression and Processing; p53-Dependent G1 DNA Damage Response; Transcriptional activation of p53 responsive genes ; The role of GTSE1 in G2/M progression after G2 checkpoint; DNA Repair; Regulation of PTEN gene transcription; Protein folding; Hemostasis; DNA Double-Strand Break Repair; Apoptosis; Transcriptional regulation by RUNX3; Cell Cycle; Cellular responses to stress; Transcriptional Regulation by TP53; Pre-NOTCH Transcription and Translation |
| UGT1A1 | NM_000463.2 , c.1411G>A , p.(Ala471Thr) | missense_variant | Gene Name: UDP-glucuronosyltransferase 1A1; UGT1A1, Family/Subfamily: UDP-GLUCURONOSYLTRANSFERASE 1A1 (PTHR48050:SF8), Protein Class: glycosyltransferase(PC00111), Pathway: Nicotine degradation->UDP glycosytransferase 1, polypeptide A4; ; , Reactome Pathway: Phase II - Conjugation of compounds; Metabolic disorders of biological oxidation enzymes; Disease; Glucuronidation; Defective UGT1A1 causes hyperbilirubinemia; Biological oxidations; Diseases of metabolism; Metabolism |
| UGT1A10 | NM_019075.2 , c.1402G>A , p.(Ala468Thr) | missense_variant | Gene Name: UDP-glucuronosyltransferase 1A10; UGT1A10, Family/Subfamily: UDP-GLUCURONOSYLTRANSFERASE 1A10-RELATED (PTHR48050:SF6), Protein Class: glycosyltransferase(PC00111), Reactome Pathway: Phase II - Conjugation of compounds; Glucuronidation; Biological oxidations; Metabolism |
| UGT1A3 | NM_019093.2 , c.1414G>A , p.(Ala472Thr) | missense_variant | Gene Name: UDP-glucuronosyltransferase 1A3; UGT1A3, Family/Subfamily: UDP-GLUCURONOSYLTRANSFERASE 1A3-RELATED (PTHR48050:SF12), Protein Class: glycosyltransferase(PC00111), Pathway: Nicotine degradation->UDP glycosytransferase 1, polypeptide A4; ; , Reactome Pathway: Phase II - Conjugation of compounds; NR1H2 & NR1H3 regulate gene expression to control bile acid homeostasis; Signaling by Nuclear Receptors; NR1H2 and NR1H3-mediated signaling; Glucuronidation; Signal Transduction; Biological oxidations; Metabolism |
| UGT1A4 | NM_007120.2 , c.1414G>A , p.(Ala472Thr) | missense_variant | Gene Name: UDP-glucuronosyltransferase 1A4; UGT1A4, Family/Subfamily: UDP-GLUCURONOSYLTRANSFERASE 1A3-RELATED (PTHR48050:SF12), Protein Class: glycosyltransferase(PC00111), Pathway: Nicotine degradation->UDP glycosytransferase 1, polypeptide A4; ; , Reactome Pathway: Metabolism; Metabolism of porphyrins; Phase II - Conjugation of compounds; Metabolic disorders of biological oxidation enzymes; Biological oxidations; Diseases of metabolism; Disease; Heme degradation; Glucuronidation; Defective UGT1A4 causes hyperbilirubinemia |
| UGT1A5 | NM_019078.1 , c.1414G>A , p.(Ala472Thr) | missense_variant | Gene Name: UDP-glucuronosyltransferase 1A5; UGT1A5, Family/Subfamily: UDP-GLUCURONOSYLTRANSFERASE 1A3-RELATED (PTHR48050:SF12), Protein Class: glycosyltransferase(PC00111), Pathway: Nicotine degradation->UDP glycosytransferase 1, polypeptide A4, Reactome Pathway: Phase II - Conjugation of compounds; Glucuronidation; Biological oxidations; Metabolism |
| UGT1A6 | NM_001072.3 , c.1408G>A , p.(Ala470Thr) | missense_variant | Gene Name: UDP-glucuronosyltransferase 1-6; UGT1A6, Family/Subfamily: UDP-GLUCURONOSYLTRANSFERASE 1-6 (PTHR48050:SF7), Protein Class: glycosyltransferase(PC00111), Pathway: Phase II - Conjugation of compounds; Glucuronidation; Biological oxidations; Metabolism |
| UGT1A7 | NM_019077.2 , c.1402G>A , p.(Ala468Thr) | missense_variant | Gene Name: UDP-glucuronosyltransferase 1A7; UGT1A7, Family/Subfamily: UDP-GLUCURONOSYLTRANSFERASE 1A10-RELATED (PTHR48050:SF6), Protein Class: glycosyltransferase(PC00111), Reactome Pathway: Phase II - Conjugation of compounds; Glucuronidation; Biological oxidations; Metabolism |
| UGT1A8 | NM_019076.4 , c.1402G>A , p.(Ala468Thr) | missense_variant | Gene Name: UDP-glucuronosyltransferase 1A8; UGT1A8, Family/Subfamily: UDP-GLUCURONOSYLTRANSFERASE 1A10-RELATED (PTHR48050:SF6), Protein Class: glycosyltransferase(PC00111), Reactome Pathway: Phase II - Conjugation of compounds; Glucuronidation; Biological oxidations; Metabolism |
| UGT1A9 | NM_021027.2 , c.1402G>A , p.(Ala468Thr) | missense_variant | Gene Name: UDP-glucuronosyltransferase 1A9; UGT1A9, Family/Subfamily: UDP-GLUCURONOSYLTRANSFERASE 1A10-RELATED (PTHR48050:SF6), Protein Class: glycosyltransferase(PC00111), Pathway: Phase II - Conjugation of compounds; Glucuronidation; Metabolism of lipids; PPARA activates gene expression; Biological oxidations; Regulation of lipid metabolism by PPARalpha; Metabolism |
| VPS33B | NM_018668.4 , c.944G>A , p.(Arg315Gln) | missense_variant | Gene Name: Vacuolar protein sorting-associated protein 33B; VPS33B, Family/Subfamily: VACUOLAR PROTEIN SORTING-ASSOCIATED PROTEIN 33B (PTHR11679:SF1), Protein Class: membrane trafficking regulatory protein(PC00151), GO-Slim Biological Process: vesicle-mediated transport(GO:0016192); intracellular protein transport(GO:0006886), Reactome Pathway: Prevention of phagosomal-lysosomal fusion; Response of Mtb to phagocytosis; Disease; Suppression of phagosomal maturation; Infectious disease; Infection with Mycobacterium tuberculosis |
| WDR36 | NM_139281.2 , c.1973A>G , p.(Asp658Gly) | missense_variant | Gene Name: WD repeat-containing protein 36; WDR36, Family/Subfamily: WD REPEAT-CONTAINING PROTEIN 36 (PTHR22840:SF12), GO-Slim Biological Process: rRNA processing(GO:0006364), Reactome Pathway: Metabolism of RNA; rRNA processing in the nucleus and cytosol; rRNA processing; rRNA modification in the nucleus and cytosol; Major pathway of rRNA processing in the nucleolus and cytosol |
| WNK1 | NM_213655.4 , c.2147G>A , p.(Arg716His) | missense_variant | Gene Name: Serine/threonine-protein kinase WNK1; WNK1, Family/Subfamily: SERINE/THREONINE-PROTEIN KINASE WNK1 (PTHR13902:SF46), Protein Class: non-receptor serine/threonine protein kinase(PC00167), GO-Slim Molecular Function: chloride channel activity(GO:0005254); protein serine/threonine kinase activity(GO:0004674); potassium channel activity(GO:0005267); potassium channel regulator activity(GO:0015459), GO-Slim Biological Process: regulation of sodium ion transport(GO:0002028); potassium ion transmembrane transport(GO:0071805); negative regulation of transport(GO:0051051); ion homeostasis(GO:0050801); sodium ion transmembrane transport(GO:0035725); regulation of potassium ion transmembrane transport(GO:1901379); positive regulation of ion transmembrane transporter activity(GO:0032414); protein phosphorylation(GO:0006468); intracellular signal transduction(GO:0035556), Reactome Pathway: Stimuli-sensing channels; Ion channel transport; Transport of small molecules |
| ZNF44 | NM_001164276.1 , c.1109G>A , p.(Gly370Glu) | missense_variant | Gene Name: Zinc finger protein 44; ZNF44, Family/Subfamily: ZINC FINGER PROTEIN 44 (PTHR24379:SF98), Protein Class: C2H2 zinc finger transcription factor(PC00248), GO-Slim Molecular Function: RNA polymerase II transcription regulatory region sequence-specific DNA binding(GO:0000977); DNA-binding transcription factor activity, RNA polymerase II-specific(GO:0000981), GO-Slim Biological Process: transcription by RNA polymerase II(GO:0006366); regulation of transcription by RNA polymerase II(GO:0006357) |

Only variants with a possible/probable damaging PolyPhen effect were included, as per data annotated by BaseSpace or detected manually using PolyPhen-2 Wiki.
