# Supplementary material for: The anterior gradient homologue 2 (AGR2) co-localises with the glucose-regulated protein 78 (GRP78) in cancer stem cells, and is critical for the survival and drug resistance of recurrent glioblastoma: in situ and in vitro analyses
Source: Cancer Cell Int. 2022 Dec 8;22:387. doi: 10.1186/s12935-022-02814-5 (PMC9730595; doi:10.1186/s12935-022-02814-5)
Supplement: Supplementary file 8 — Additional file 8: Table S3. It is likely affect prominent functions in the studied cell lines. Genes with rare damaging COSMIC variants were detected in the tissue and corresponding cell line for each patient and organized by prominent functions (Panther: Molecular Function/ Biological Process/Pathway/Reactome Pathway, NCBI Gene information*) for both Jed66_GB and Jed41_GB. Only variants with a possible/probable damaging PolyPhen effect were included, as per data annotated by BaseSpace or as detected manually using PolyPhen-2 Wiki. [file 12935_2022_2814_MOESM8_ESM.docx]

**Supplementary Table 3.** Likely to be affected prominent functions in the studied cell lines. Genes with rare damaging COSMIC variants detected in the tissue and corresponding cell line for each patient, and organized by prominent functions for both Jed66_GB and Jed41_GB.

| **Prominent Function** | **Jed66_GB** | **Jed41_GB** |
| --- | --- | --- |
| **Transcription** | ARID1B: Chromatin organization; NCOR1:Transcription repressor; NFE2L2: Positive regulation of transcription | ATN1: Regulation of PTEN gene transcription; CASP8AP2: Transcription corepressor; MAZ: DNA-binding transcription factor; NFE2L2: DNA-binding transcription factor; RCOR3: Transcription corepressor; TP53: Critical transcription factor; ZNF44: C2H2 zinc finger transcription factor |
| **Membrane trafficking** | DENND3: Rab regulation of trafficking; ACAP1: GTPase activator activity and metal ion binding activity; CD48: Cell surface interactions; EPN2: clathrin-mediated endocytosis; FADS6: predicted to be integral component of membrane*; KIF1A: Vesicle-mediated transport; MB21D2: Enables cadherin binding activity*; OTOP1: Proton channel transporter; POTEH: Predicted to be integral component of membrane*; RNF225: Predicted to enable metal ion binding activity*; TBC1D26: Positive regulation of GTPase activity; TRARG1: Endosome to plasma membrane protein transport and glucose import in response to insulin stimulus* | LRP1B: Low-density lipoprotein receptor-related protein; FADS6: Lipid metabolic and is an integral component of membrane*; KCNK17: Potassium ion transmembrane transport; STAB2: Regulatory protein; VPS33B: a membrane trafficking regulator; WNK1: ion channel transport |
| **Signalling pathway** | BCR: FGFR1 mutant receptor activation; CRIPAK: Negative regulator of PAK1*; LRIG3: Transmembrane signal receptor;  LTB4R: G alpha (q) signalling events; MADCAM1: integrin-mediated signalling pathway; OR51B2: G alpha (s) signalling; PTPN5: Interleukin-37 signalling; SELP: Signalling and aggregation; SSTR3: G alpha (i) signalling; TYK2: type I and type III interferon signalling pathways | ADGRE5: Regulation of cAMP-mediated signalling; ADGRF4: a member of the superfamily of G protein-couple receptors*; OR2T11: G alpha (s) signalling; OR2T33: G alpha (s) signalling; OR3A1: G alpha (s) signalling |
| **Cell cycle and division** | SEPT6: Cytoskeleton-dependent cytokinesis; SUSD2: Involved in negative regulation of cell cycle G1/S phase transition and negative regulation of cell division | CCNF: Mitotic cell cycle phase transition; LNP1: Important for nuclear pore complexes*; MISP3: Spindle orientation and mitotic progression*; SUN2: Nuclear envelope organization; SUSD2: Negative regulation of cell cycle G1/S phase transition* |
| **Post-translational protein modification** | ABHD17A: Lipoprotein metabolic process; ARSD: Gamma carboxylation; DIS3L: Effects methylation*; FAM214B: 3'-5'-exoribonuclease activity; MELTF: Regulation of Insulin-like Growth Factor (IGF) transport and uptake by Insulin-like Growth Factor Binding Proteins (IGFBPs); MUC2: O-glycan biosynthesis; MUC3A: O-glycan biosynthesis; STC2: Regulation of Insulin-like Growth Factor (IGF); PLA2G4D: glycerophospholipid biosynthesis; PRSS3: Metabolism of vitamins and cofactors; USP18: deubiquitination | FN3K: Gamma carboxylation; HDDC3: Phosphoric ester hydrolase activity; LIPF: Gastric lipase; MUC2: O-glycan biosynthesis; MUC3A: O-glycan biosynthesis; PHLDB3: Enables enzyme binding activity; PRSS45P: Serine-type endopeptidase activity; WDR36: rRNA processing |
| **Glycolysis** | SORD: Fructose biosynthesis; TPI1: Glycolysis metabolism | CERCAM: UDP-glycosyltransferase activity; UGT1A1:Glycosyltransferase; UGT1A10: Glycosyltransferase; UGT1A3: Glycosyltransferase; UGT1A4: Glycosyltransferase; UGT1A5 Glycosyltransferase: ; UGT1A6: Glycosyltransferase; UGT1A7: Glycosyltransferase; UGT1A8: Glycosyltransferase; UGT1A9: Glycosyltransferase |
| **Intercellular structure and movement** | CDC42BPA: Actomyosin organization; CFAP100: Predicted to enable dynein complex binding activity*; KIF26B: COPI-dependent Golgi-to-ER retrograde traffic; LNP1: Important for nuclear pore complexes*; NPHP3: Cargo trafficking to the periciliary membrane; PHLDB1: Regulation of microtubule cytoskeleton organization*; RP1L1: Regulate microtubule polymerization* | None |
| **Cellular responses to stress** | DNAJA4: HSP90 chaperone cycle for steroid hormone receptors (SHR); TRAP1: ATPase activity and interacts with tumour necrosis factor type I* | None |
| **Chromosome stability** | PCNX2: High microsatellite instability (MSI-H)*; POLD1: mismatch repair directed by MSH2:MSH3 | None |
| **Other functions** | None | ATG2B : Endoplasmic reticulum organization; CCDC40: Motile cilia function*; DMXL1: Vacuolar acidification; FAM187B: Immunoglobulin superfamily cell adhesion |

Only variants with a possible/probable damaging PolyPhen effect were included, as per data annotated by BaseSpace or as detected manually using PolyPhen-2 Wiki. Asterisk indicate information retrieved from Panther Molecular Function/ Biological Process/Pathway/ Reactome Pathway, NCBI Gene information.
